# Enzyme‐Activated MRI for In Vivo Glucose Imaging via a Biodegradable Chromium Nanoprobe

**DOI:** 10.1002/advs.74861

**Published:** 2026-03-15

**Authors:** Yan Xu, Weitao Yang, Yanjing Yun, Hui Wang, Zhuoyao Wu, Youyi Yu, Bingbo Zhang

**Affiliations:** ^1^ Department of Radiology Tongji Hospital Shanghai Frontiers Science Center of Nanocatalytic Medicine the Institute for Biomedical Engineering & Nano Science School of Medicine Tongji University Shanghai China; ^2^ Shanghai Key Laboratory of Molecular Imaging Shanghai Pudong New Area Gongli Hospital Affiliated Shanghai University of Medicine and Health Sciences Shanghai China

**Keywords:** biomimetic synthesis, catalysis imaging, chromium‐based MR probes, glucose imaging, responsive MRI

## Abstract

Non‐invasive visualization of glucose metabolism in living organisms remains a major challenge, as existing techniques cannot specifically detect glucose molecules with deep‐tissue penetration and without ionizing radiation. To overcome this, we developed an enzyme‐activated magnetic resonance imaging strategy (eaMRI) using a biodegradable nanoprobe, CrGOx@Lip, that integrates endogenous Cr^3+^ ions with glucose oxidase for glucose‐responsive imaging. Our approach leverages GOx not only as a catalytic engine to specifically oxidize glucose but also as a structural template to guide the in‐situ synthesis of a potent MRI reporter (paramagnetic chromium gluconate). This design enables direct, specific amplification of MRI signals in proportion to local glucose concentration, achieving an 8.28‐fold relaxivity increase. We validated this method for sensitive glucose mapping in vivo, including delineating Warburg‐effect‐driven tumors and quantifying pathological glucose accumulation in metabolic dysfunction‐associated fatty liver disease (ΔSNR% = 18.51 ± 3.72 vs. 1.14 ± 1.39 in controls; *p* <0.001), and further demonstrated its utility in monitoring therapeutic efficacy via glucose‐responsive signal changes. By synergizing enzymatic precision with nanomaterial engineering, our CrGOx@Lip‐mediated eaMRI platform provides a glucose‐specific, radiation‐free, and non‐invasive strategy for sensitive metabolic diagnostics in precision medicine.

## Graphical abstract

Enzyme‐activated MRI (eaMRI) using a biodegradable CrGOx@Lip nanoprobe enables glucose‐specific imaging in vivo. GOx catalyzes glucose oxidation, triggering in situ formation of paramagnetic chromium gluconate and amplifying *T*_1_. This radiation‐free strategy maps tumor glucose uptake via the Warburg‐effect, quantifies hepatic glucose accumulation in MAFLD, and evaluates therapy response of metabolic interventions.

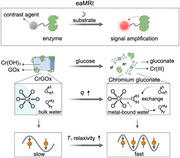

## 1 Introduction

Glucose is a pivotal biomarker across cancer, metabolic disorders, and liver disease [1, 2, 3], yet non‐invasive imaging of glucose in vivo remains fundamentally limited [4, 5, 6]. Although tumors exploit aerobic glycolysis (Warburg effect) and metabolic dysfunction‐associated fatty liver disease (MAFLD) is characterized by pathological hepatic glucose accumulation [7, 8, 9], current modalities fail to combine molecular specificity, adequate tissue depth, and radiation‐free safety. Invasive sensors risk infection, while optical probes are restricted by poor tissue penetration [10, 11]. ^18^F‐FDG PET—although sensitive and clinically established as the gold standard for glucose metabolic imaging—involves exposure to ionizing radiation and, as a glucose analog, cannot directly distinguish glucose itself from its structural analogs, leading to potential false positives in inflammatory conditions [12, 13]. Consequently, there is an urgent demand for glucose‑specific, high‑penetration, non‑radioactive imaging strategies capable of assessing glucose metabolism in complex biological environments.

Magnetic resonance imaging (MRI) is a highly promising approach for metabolic imaging, as it provides exceptional spatial resolution along with non‐invasive capabilities and effective tissue penetration [14, 15, 16]. However, conventional structural MRI is not specific to directly detect glucose. Similarly, functional MRI (fMRI) and magnetic resonance spectroscopy (MRS) have their own limitations [17, 18, 19]. Although MRS has made significant progress in identifying small metabolites, its clinical applications are restricted due to issues such as low specificity, inadequate reproducibility, and restricted spatial resolution [20, 21, 22]. Chemical exchange saturation transfer (CEST) approaches such as glucoCEST have pioneered glucose‐derived contrast by exploiting the exchangeable protons of sugar. Nevertheless, they remain limited by inherently low sensitivity at clinical field strengths below 3 T, prolongated acquisition times, and susceptibility to interference from endogenous metabolites [23, 24, 25]. Together, these limitations highlight an urgent need for molecular MRI probes that deliver both high sensitivity and high specificity for glucose imaging.

**FIGURE 1:**
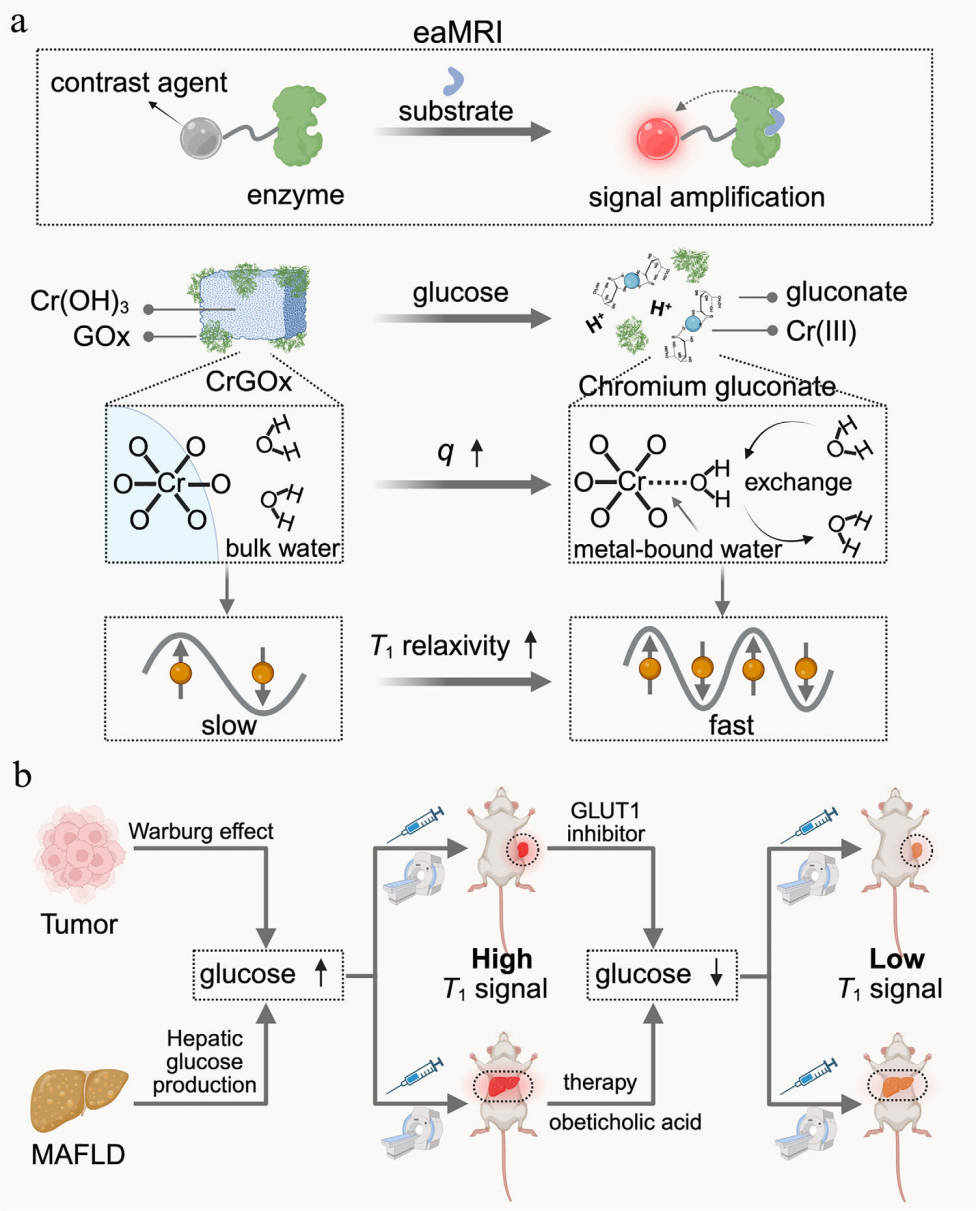
Illustration of eaMRI strategy and its CrGOx probe for glucose‐specific MRI. (a) Schematic diagram of the eaMRI work: converting enzymatic reaction events into detectable magnetic resonance signals; and glucose‐specific *T*_1_‐enhancing mechanism of CrGOx‐mediated eaMRI (*q*: the hydration number). (b) In vivo real‐time glucose mapping in solid tumors and hepatic glucose accumulation in MAFLD. Created with BioRender.com.

Building upon the challenges of imaging small metabolites in vivo, such as structural similarity among endogenous analogs, rapid diffusion, and endogenous interference, we developed enzyme‐activated magnetic resonance imaging (eaMRI), a molecular strategy that leverages the specificity and catalytic power of enzyme‐substrate interactions to overcome key limitations in conventional imaging. To implement eaMRI, a key challenge lies in converting enzymatic catalytic events into detectable MRI signals. Here, eaMRI uniquely integrates glucose oxidase (GOx) catalysis with nanomaterial‐enabled signal amplification (Figure 1a). Our GOx‐engineered chromium nanoprobe (CrGOx) exploits GOx's dual role as a biotemplate and enzymatic activator. Upon glucose exposure, the hydrogen ions (H^+^) released during the GOx‑catalyzed oxidation dissolves biomineralized Cr(OH)_3_ to form paramagnetic chromium gluconate in situ—a dietary supplement chelate that retains high relaxivity while eliminating free‐ion toxicity risks, amplifying *T*_1_ contrast (> 8‐fold relaxivity enhancement at physiological glucose levels) with high specificity. Crucially, liposomal encapsulation (CrGOx@Lip) prevents premature activation while enabling real‐time mapping of tumor glucose uptake via Warburg‐effect targeting, quantification of hepatic glucose accumulation in MAFLD (closely linked to insulin resistance), and therapy response evaluation for metabolic interventions (Figure 1b). Validated in both tumor and MAFLD models, this work establishes eaMRI as a molecular imaging platform for metabolic MRI, offering radiation‑free operation and direct glucose specificity to complement PET and CEST for precision diagnostics.

## 2 Results and Discussion

### 2.1 Enzyme‐Activated MRI (eaMRI): Strategy and Preparation of the CrGOx Probe

To implement eaMRI, we designed and fabricated responsive probes capable of transforming enzymatic reaction energy within targeted regions into quantifiable MRI readouts. Specifically, we employed GOx to catalyze the oxidation of glucose into gluconate and release H^+^. This reaction was strategically coupled with an acid‐sensitive Cr(OH)_3_ matrix, which dissolves in the local acid condition to form paramagnetic chromium gluconate in situ—generating a strong MRI *T*_1_ signal.

**FIGURE 2:**
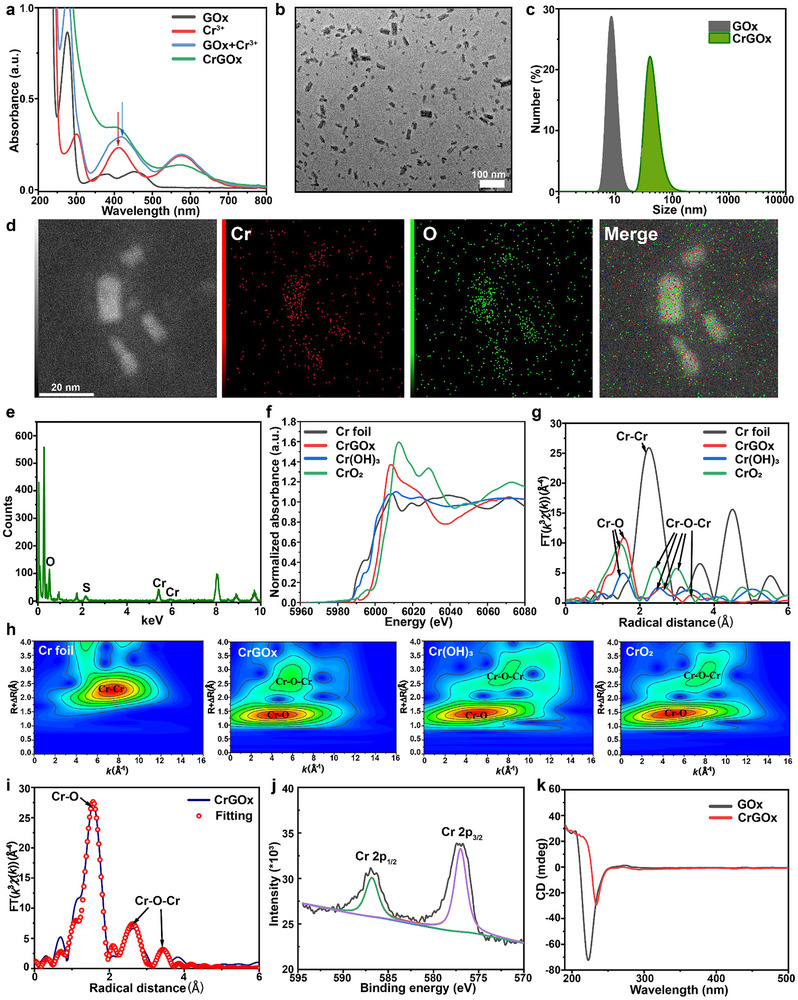
Characterization of CrGOx. (a) UV–vis absorption spectra of GOx, Cr^3+^ ions from Cr(NO_3_)_3_, a physical mixture of GOx and Cr^3+^ (GOx+ Cr^3+^), and the synthesized CrGOx. (b) TEM image of CrGOx (scale bar: 100 nm). (c) Dynamic light scattering (DLS) analyses of GOx and CrGOx. (d) Element mapping of CrGOx (scale bar: 20 nm). (e) EDS spectrum of CrGOx. (f) XANES spectra at Cr K‐edge. (g) FT‐EXAFS spectra. (h) WT plot of Cr K‐edge signal of Cr foil, CrGOx, Cr(OH)_3_, and CrO_2_. (i) Fitting curves of EXAFS spectra for Cr at R space. (j) XPS spectrum (Cr 2p) spectrum of CrGOx. (k) circular dichroism spectroscopy of free GOx and CrGOx.

Capitalizing on this mechanism, we constructed a hybrid probe through GOx‐templated biomineralization [26, 27, 28], designated CrGOx, for sensitive and specific glucose imaging. Cr^3+^ was selected as the MRI contrast element because it is a paramagnetic ion capable of accelerating longitudinal proton relaxation [29, 30]. The relaxivity of Cr^3+^ is highly related to its coordination environment—particularly the number of inner‑sphere water molecules and the water exchange kinetics. Additionally, it is an essential trace element for the human body, playing important roles in glucose and lipid metabolism regulation [31]. GOx served as both a biotemplate and chelating scaffold, enabling Cr^3+^ coordination from Cr(NO_3_)_3_ into uniform nanoparticles. As shown in Figure 2a, the ultraviolet‐visible (UV–vis) absorption shift of Cr^3+^ from 410 to 418 nm upon GOx addition suggested the interaction between Cr^3+^ ions and GOx. Additionally, the appearance of characteristic absorption peaks at 403 and 576 nm in CrGOx confirmed the incorporation of Cr^3+^ into the CrGOx structure. Transmission electron microscopy (TEM) result (Figure 2b) revealed a rectangular structure of CrGOx with an average diameter of about 26 nm. Dynamic light scattering (DLS) measurements (Figure 2c) indicated a hydrodynamic diameter of approximately 44 nm, slightly larger than the TEM‐measured size. This difference can be explained by the presence of the GOx‐enriched outer layer that contributes to the hydrodynamic size in aqueous solution. Zeta potential of CrGOx was measured at ‐11.9 mV (Figure S1). Control synthesis without GOx yielded aggregates (234 nm; Figure S2), underscoring the critical role of GOx in synthesis. Additionally, hydrodynamic size and the relaxation times of CrGOx were monitored over 7 days. The results (Figure S3) show that the particle size remains around 40 nm, and the *T*_1_ and *T*_2_ values of CrGOx remain consistent with minimal variations, indicating good stability.

Element mapping results (Figure 2d) revealed a uniform distribution of chromium and oxygen within CrGOx nanoparticles, which was further confirmed by energy‐dispersive spectroscopy (EDS) (Figure 2e) and X‐ray photoelectron spectroscopy (XPS) compositional analysis (Figure S4). The chemical state and coordination environment of Cr was investigated by using the X‐ray absorption fine structure (XAFS) analysis. The Cr K‐edge X‐ray absorption near edge structure (XANES) analysis (Figure 2f) revealed that the pre‐edge position of CrGOx is intermediate between those of the Cr(OH)_3_ and CrO_2_ reference standards, suggesting an oxidation state of Cr in CrGOx that lies between +3 and +4. Fourier‐transformation extended X‐ray absorption fine structure (FT‐EXAFS) spectra (Figure 2g) revealed a dominant peak at 1.57 Å, which can be designated as the Cr─O scattering path in the first coordination sphere. Meanwhile, weak peaks at 2.64 and 3.41 Å observed in the CrGOx spectrum exhibited close correspondence with those observed in the Cr(OH)_3_ reference standard, which can be attributed to multiple Cr─O─Cr scattering pathways within the second coordination shell. These findings are confirmed by the wavelet transform (WT) plot (Figure 2h). EXAFS analysis, detailed in Figure 2i, Figures S5 and S6, and Table S1, revealed that the Cr center exhibits an average Cr─O coordination number of 5.2 ± 0.2 with a bond length of 1.98 Å. This strong and sufficient coordination with oxygen‐donor ligands suppressed proton relaxation rate, as will be addressed in subsequent mechanistic discussions. Furthermore, XPS analysis of the chemical state of Cr (Figure 2j) showed binding energy peaks at 577.0 eV (2p_3/2_) and 586.8 eV (2p_1/2_), which are characteristic of Cr^3+^ in Cr(OH)_3_. These results collectively identify that the primary form of chromium in CrGOx is Cr(OH)_3_.

X‐ray diffraction (XRD) analysis (Figure S7) and selected area electron diffraction (SAED) pattern (Figure S8) revealed that CrGOx have an amorphous structure. Circular dichroism (CD) spectroscopy revealed alterations in GOx's secondary structure within the nanocomposite where the characteristic negative peak redshift from 222 to 234 nm (Figure 2k), accompanied by a 59.9% reduction in peak intensity. Crucially, enzymatic activity assays demonstrated that CrGOx preserved 39.2% of its native catalytic capacity of GOx relative to unmodified GOx (Figure S9), confirming functional integrity despite structural reorganization. Collectively, these findings establish CrGOx as a structurally well‐defined, enzymatically active nanocomposite engineered for responsive imaging.

### 2.2 Glucose‐Responsive Relaxation Properties of CrGOx

**FIGURE 3:**
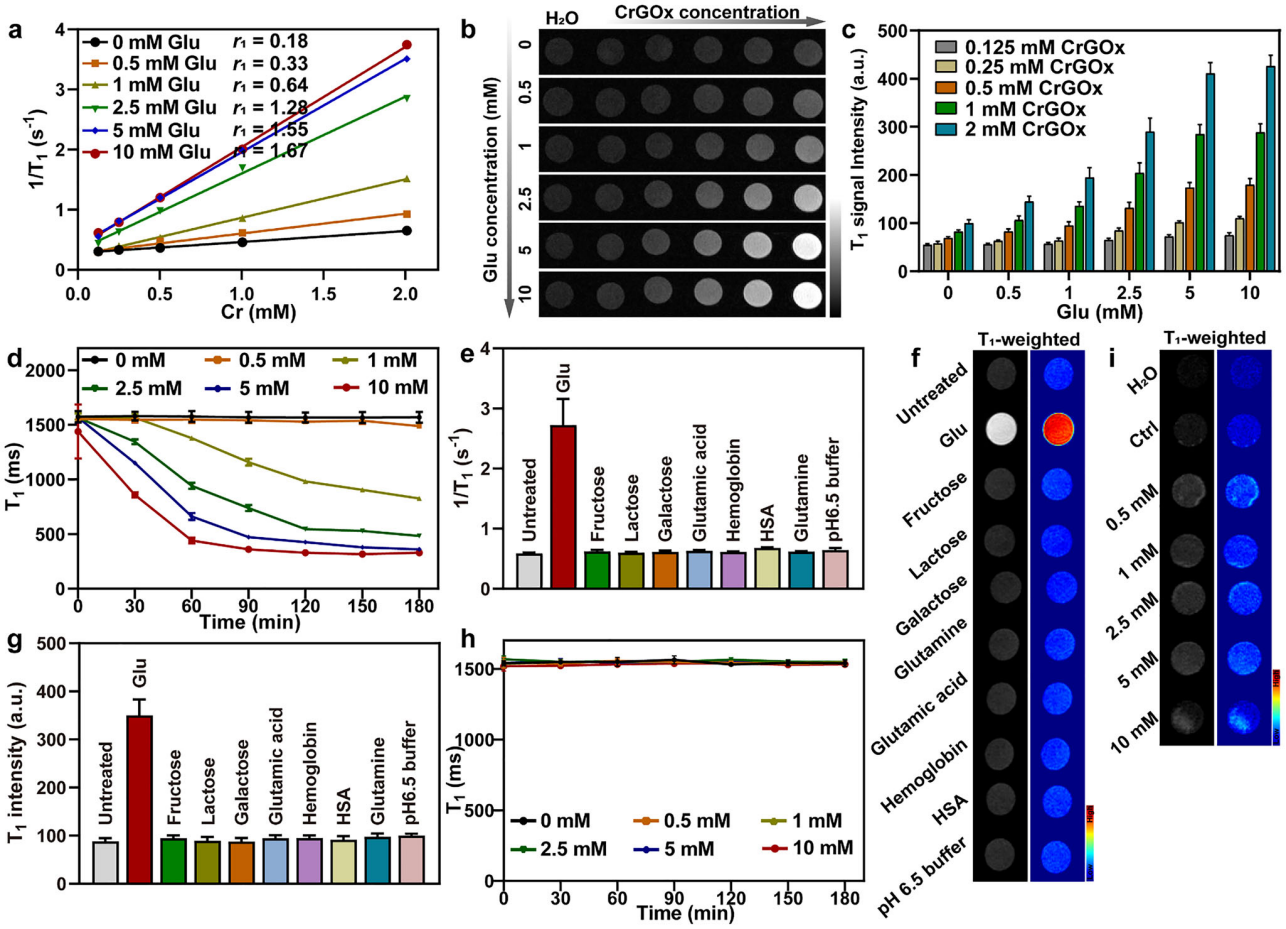
Glucose‐responsive relaxation properties of CrGOx. (a) The longitudinal relaxation curve fitting, (b) in vitro *T*_1_‐weighted MRI, and (c) signal intensity of CrGOx across varying glucose concentrations. (d) *T*_1_ relaxation time of CrGOx over time across varying glucose concentrations. (e) *T*_1_ relaxation time, (f) in vitro *T*_1_‐weighted MRI, and (g) signal intensity of CrGOx with glucose, fructose, lactose, galactose, glutamine, glutamic acid, hemoglobin, human serum albumin (HSA), and pH 6.5 buffer at concentration of 10 mm. (h) *T*_1_ relaxation time and (i) *T*_1_‐weighted MRI of CrBSA across varying glucose concentrations.

CrGOx demonstrated pronounced glucose‐dependent relaxivity modulation, validating its design as an enzyme‐activated MRI probe. Initial longitudinal relaxivity (*r*_1_) of CrGOx was as low as 0.18 mm ^−1^·s^−1^ but increased 8.28‐fold to 1.67 mm ^−1^·s^−1^ at 10 mm glucose, exhibiting glucose concentration‐dependent amplification (Figure 3a). Correspondingly, *T*_1_‐weighted MRI signal intensity of CrGOx showed glucose‐dependent enhancement across 0–10 mm (Figure 3b,c), with quantitative analysis (Figure 3c) revealing a 3.28‐fold increase at 10 mm glucose (2 mm CrGOx) compared to the no glucose group. Additionally, CrGOx in fetal bovine serum (FBS) exhibits similar glucose‐responsive *T*_1_ relaxation behavior (Figure S10), suggesting that CrGOx can maintain good glucose‐responsive performance in vivo. Transverse relaxivity (*r*_2_) remained minimally perturbed, increasing only marginally from 0.70 to 2.12 mm ^−1^·s^−1^ at 10 mm glucose (Figure S11), yielding an optimal *r*_2_/*r*_1_ ratio of 1.27—facilitating *T*_1_‐weighted imaging.

Kinetic studies revealed rapid activation of CrGOx toward glucose. *T*_1_ relaxation time of CrGOx was found decreased sharply within 60 min at 10 mm glucose (Figure 3d), while remaining stable without glucose exposure. Specificity screening further confirmed exclusive response to glucose. Among interferents (fructose, lactose, galactose, glutamine, glutamic acid, H_2_O_2_, hemoglobin, human serum albumin, pH 6.5 buffer), only glucose induced significant 1/*T*_1_ increase (Figure 3e). This selective response pattern was validated through *T*_1_‐weighted imaging (Figure 3f) and quantitative signal intensity analysis (Figure 3g). To verify the catalytic function of GOx in CrGOx, we synthesized a GOx‐free probe, CrBSA, using bovine serum albumin (BSA) as a template. Control experiments with CrBSA showed no glucose response (Figure 3h,i), unequivocally attributing CrGOx's activation to GOx‐mediated enzymatic recognition.

### 2.3 Mechanism of Glucose‐Responsive Relaxivity

**FIGURE 4:**
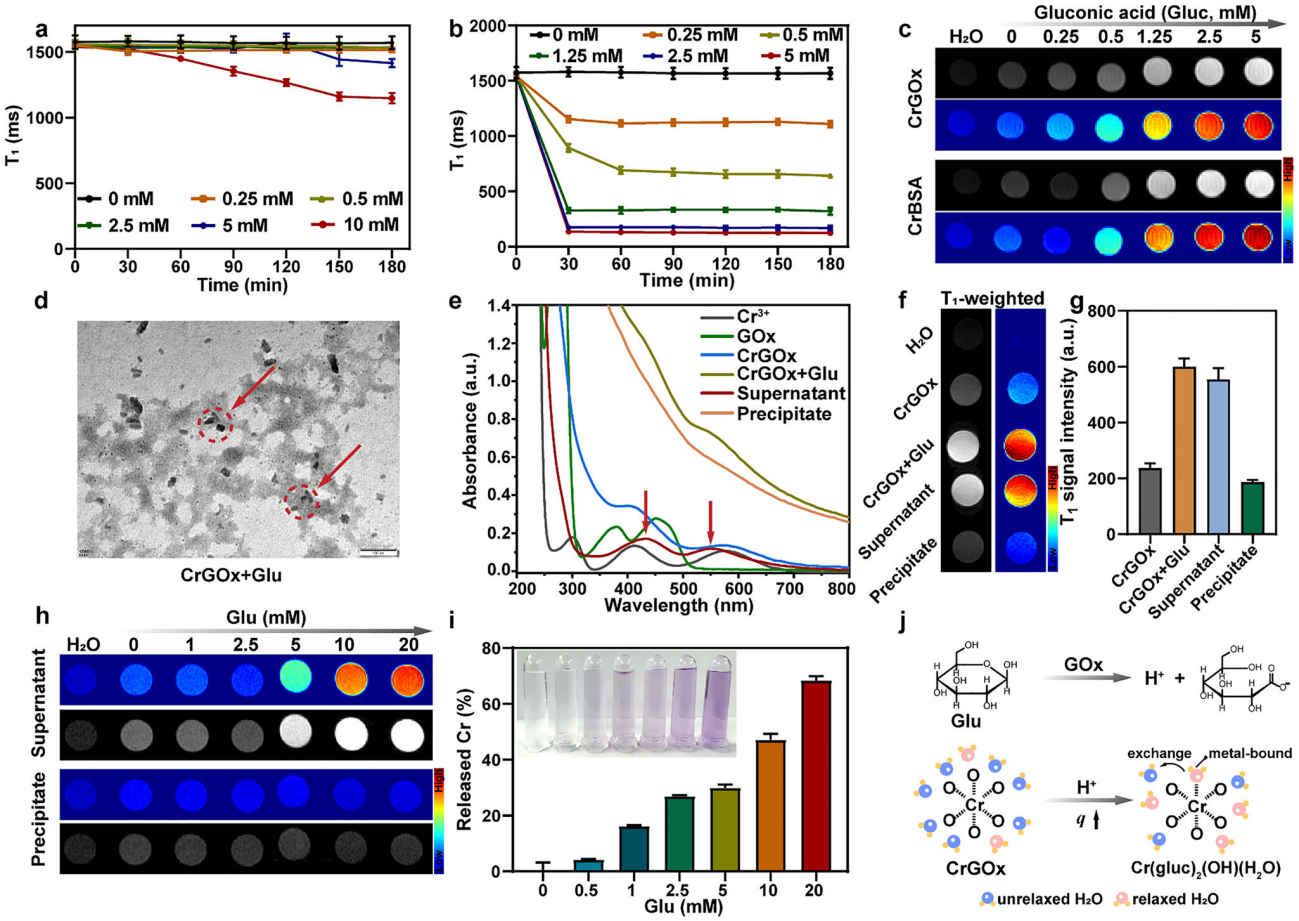
Glucose‐responsive mechanism study for CrGOx. *T*_1_ relaxation time of CrGOx across varying (a) H_2_O_2_ and (b) gluconic acid concentrations. (c) In vitro *T*_1_ MRI of CrGOx and CrBSA across varying gluconic acid concentrations. (d) TEM image of CrGOx after the reaction with glucose (the arrow points to the unreacted portion of CrGOx). (e) UV–vis absorption spectra of Cr^3+^, GOx, CrGOx, CrGOx+Glu mixture, the supernatant, and the precipitate. (f) In vitro *T*_1_ MRI, and (g) signal intensity of CrGOx, CrGOx+Glu mixture, the supernatant, and the precipitate. (h) In vitro *T*_1_ MRI of the supernatant, and the precipitate across varying glucose concentrations. (i) Content of Cr^3+^ in the supernatant tested by a method of EDTA chelation and the corresponding digital picture. (j) an illustration of the proposed mechanism for glucose‐responsive *T*_1_ signal enhancement of CrGOx.

Structural analysis established Cr(OH)_3_ as the primary chromium species in CrGOx. Given its amphoteric properties, we hypothesized that the lowering of local pH—from the H^+^ released during the GOx‑catalyzed oxidation—mediates acid dissolution of the Cr(OH)_3_ core to paramagnetic chelates. To validate this mechanism, we decoupled catalytic products. H_2_O_2_ exposure (0‐5 mm) to CrGOx induced negligible changes in *T*_1_ relaxation time over 180 min (Figure 4a), whereas gluconic acid (5 mm) triggered rapid *T*_1_ reduction from 154 ms to 124 ms within 30 min (Figure 4b). Additionally, Cr^3+^ ions incubated with H_2_O_2_ (0–100 mm) for 3 h showed no significant difference in *T*_1_ signal (Figure S12), indicating that CrGOx does not undergo a Fenton‐like reaction affecting *T*_1_ relaxation signal. In vitro MRI assays revealed that both CrGOx and GOx‐free CrBSA exhibited acid‐dependent signal amplification (Figure 4c; Figure S13). To clarify whether the signal amplification is driven by the lowering of local pH or by gluconate, we performed additional experiments. When CrGOx was incubated with sodium gluconate (pH 7.4 adjusted with NaOH), no significant change in *T*_1_‑weighted MRI signal was observed compared to CrGOx alone (Figure S14). In contrast, incubation with hydrochloric acid (HCl) resulted in a pronounced increase in *T*_1_ signal. The same pattern was observed with the control nanoparticle CrBSA, confirming that the response is primarily driven by acidic conditions rather than by gluconate.

Post‐glucose structural characterization revealed partial nanoparticle degradation of CrGOx (Figure 4d). UV–vis absorption spectra of supernatant fractions exhibited characteristic Cr^3+^ absorption peaks at 432 and 550 nm (Figure 4e), with corresponding *T*_1_‐weighted MRI signals 2.97‐fold higher than precipitates (Figure 4f,g). Glucose concentration‐dependent signal enhancement (0–20 mm) was exclusive to supernatants (Figure 4h; Figure S15). EDTA‐chelation assays quantified progressive Cr^3+^ release, escalating from 16.3% at 1 mm glucose to 68.4% at 20 mm glucose (Figure 4i; Figures S16–S18). These results indicate that the coordination form of chromium in CrGOx changes after glucose response. We further investigated the final form of chromium after catalysis. As demonstrated, free Cr^3+^ ions exhibited significantly higher *r*_1_ relaxivity (Figure S19) than CrGOx post‐glucose exposure (*r*_1_ = 5.16 vs. 1.67 mM^−1^·s^−1^), suggesting that the dissolved CrGOx didn't persist as free Cr^3+^ ions. As verified by UV–vis spectral alignment with standards (λ_max_ = 432/548 nm; Figure S20) and mass spectrometry detection of the m/z 442 ([Cr(C_6_H_11_O_7_)_2_]^−^) and m/z 638 ([Cr(C_6_H_11_O_7_)_3_‐H]^−^) molecular ion (Figure S21), the final form of chromium after catalysis was determined to be chromium gluconate. Notably, chromium gluconate is a biocompatible dietary supplement [30, 31].

Solomon‐Bloembergen‐Morgan theory echoes that while CrGOx's stable Cr─O coordination (5.2 ± 0.2, Table S1) restricts the water coordination (*q* ≈ 0) [32, 33], the formed chromium gluconate ([Cr(C_6_H_11_O_7_)_2 or 3_]^−^) after catalysis increases inner‐sphere water coordination sites (*q*≥1), explaining the 8.28‐fold *r*_1_ enhancement, escalating from 0.18 to 1.67 at 10 mm glucose. This cascade of events—enzymatic glucose oxidation, local H^+^ and gluconate release, Cr(OH)_3_ dissolution, and paramagnetic chromium gluconate formation—enables glucose‐specific MRI contrast amplification.

### 2.4 Tumor Glucose Imaging

**FIGURE 5:**
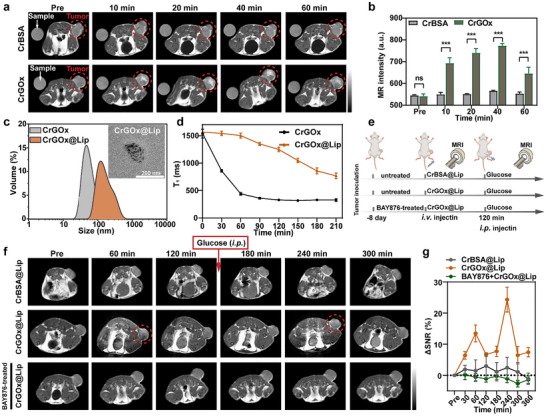
Tumor glucose imaging. (a) MRI and (b) tumor signal intensity of CT26 tumor‐bearing mice intratumorally injected with CrBSA and CrGOx. (c) DLS analysis of CrGOx and CrGOx@Lip (the inserted picture is the TEM image of CrGOx@Lip). (d) *T*_1_ relaxation time of CrGOx and CrGOx@Lip across varying glucose concentrations. (e) Schematic illustration of the animal experiment studying CrGOx@Lip for tumor glucose uptake MRI. BAY‐876 (GLUT1 inhibitor)‐treated or untreated mice were intravenously injected with CrBSA@Lip or CrGOx@Lip and intraperitoneally injected with glucose after 120 min. (f) MR images and (g) quantitative tumor signal intensity analysis of untreated mice intravenously injected with CrBSA@Lip and CrGOx@Lip, and BAY876‐pretreated mice intravenously injected with CrGOx@Lip. Data are presented as means ± SD; ^*^ *p* < 0.05, ^**^ *p* < 0.01, ^***^ *p* < 0.001; ns, no significance.

Capitalizing on the Warburg effect—where tumors preferentially uptake glucose—we evaluated CrGOx's potential for tumor glucose uptake imaging. Before intravenous administration through the tail vein, we tested the MRI performance of CrGOx via intratumoral administration. Intratumoral injection of CrGOx in CT26‐bearing mice produced a gradual signal increase that peaked at 40 min (Figure 5a,b). In contrast, control CrBSA—which contains the same acid‐responsive Cr(OH)_3_ core as GOx but lacks the enzyme—elicited no detectable response. Comparison with an ex vivo CrBSA sample placed adjacent to the mouse confirmed that intratumoral CrBSA produced a negligible signal change (Figure 5a), demonstrating that activation depends on glucose‐derived acid (pH < 3) rather than on the mildly acidic tumor microenvironment (pH ∼6.5). Moreover, the local acid condition generated at the enzyme‐nanoparticle interface creates a localized, high‐concentration acidic micro‐domain that directly dissolves chromium hydroxide and facilitates chromium gluconate formation.

To enable systemic delivery, we encapsulated CrGOx within liposomes (CrGOx@Lip; 173 nm hydrodynamic diameter, Figure 5c). Liposomal encapsulation suppressed premature activation, as evidenced by a gradual *T*_1_ decrease from 1566 to 768 ms over 210 min in the CrGOx@Lip group, whereas unencapsulated CrGOx collapsed from 1539 to 441 ms within 60 min, indicating marked inhibition of enzymatic catalysis during circulation (Figure 5d). To evaluate the mechanism‐specificity of our imaging probe, we used BAY‐876, a high‐affinity GLUT1 inhibitor, to selectively block glucose uptake in tumor cells [34]. Imaging efficacy was then compared between untreated and BAY‐876‐pretreated CT26‐bearing mice (Figure 5e). The results showed that untreated mice exhibited significant ΔSNR% enhancement (13.47 ± 2.74% at 60 min) post‐tail vein injection of CrGOx@Lip; while BAY‐876‐pretreated mice (GLUT1‐inhibited) showed negligible signal change (ΔSNR% = ‐0.59 ± 1.19%; Figure 5f,g). Notably, an intraperitoneal glucose injection at 120 min post‐probe triggered a secondary increase in tumor signal intensity at 240 min exclusively in CrGOx@Lip‐treated mice (ΔSNR% = 24.33 ± 3.98%; Figure 5f,g). This enhancement reflects further activation of residual intratumoral probe by exogenous glucose, demonstrating good capability of glucose uptake monitoring. Immunofluorescence confirmed suppressed GLUT1 in BAY‐876 groups (Figure S22), correlating with imaging specificity. Collectively, CrGOx@Lip achieves tumor‐selective glucose imaging resistant to systemic interference.

### 2.5 Hepatic Glucose Imaging in Metabolic Liver Disease

**FIGURE 6:**
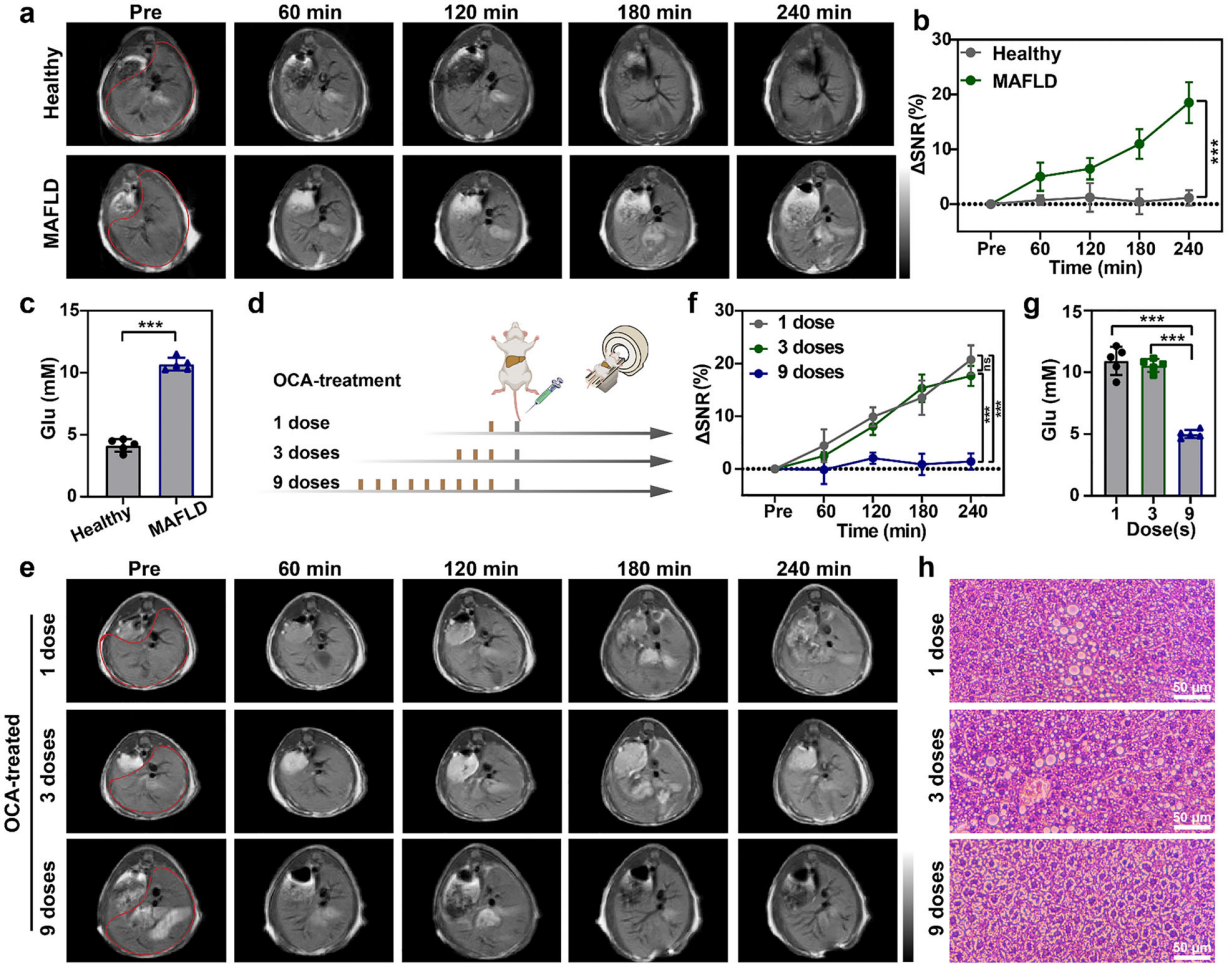
MAFLD glucose imaging. (a) MR images and (b) hepatic signal intensity analysis of healthy mice and MAFLD mice intravenously administered with CrGOx@Lip. (c) Glucose concentration in dissected liver tissue of the CrGOx@Lip‐treated healthy mice and MAFLD mice, tested by glucose assay kit. (d) Schematic illustration of the animal experiment studying CrGOx@Lip for MAFLD glucose MRI. (e) MR images and (f) hepatic signal intensity analysis of MAFLD mice pretreated with 1 dose, 3 doses, and 9 doses of OCA (a MAFLD therapeutic drug) and then intravenously administered with CrGOx@Lip. (g) glucose concentration in dissected liver tissue of the treated MAFLD mice. (h) H&E staining of livers from the OCA‐treated MAFLD mice (scale bar: 50 µm). Data are presented as means ± SD; ^*^ *p* < 0.05, ^**^ *p* < 0.01, ^***^ *p* < 0.001; ns, no significance.

Metabolic dysfunction‐associated fatty liver disease (MAFLD) features pathological hepatic glucose accumulation driven by insulin resistance and dysregulated gluconeogenesis [35, 36, 37]. We further used CrGOx@Lip to non‐invasively monitor this metabolic derangement. In MAFLD mice, it showed progressive hepatic signal enhancement (ΔSNR% = 18.51 ± 3.72% at 240 min; Figure 6a,b), while healthy controls showed negligible change (ΔSNR% = 1.14 ± 1.39%) after intravenous administration of CrGOx@Lip. This imaging contrast correlated with elevated intrahepatic glucose levels, confirmed biochemically (*p* < 0.001; Figure 6c; Figure S23) and steatosis verified by H&E staining (Figure S24), establishing CrGOx@Lip as a specific imaging tool for MAFLD‐associated metabolic dysfunction.

We further demonstrated therapeutic monitoring capability of CrGOx@Lip on obeticholic acid (OCA)‐treated MAFLD mice [38, 39, 40]. MAFLD mice receiving sub‐therapeutic OCA (1 and 3 doses) exhibited persistent CrGOx@Lip activation (ΔSNR% = 20.74% ± 2.72% and 17.66% ± 1.90%, respectively; Figure 6e,f), whereas fully treated mice (9 doses) showed significant metabolic reduction characterized by abolished *T*_1_ signal enhancement (ΔSNR% = 1.41% ± 1.53%), normalized hepatic glucose (5.01 ± 0.33 mm vs. hyperglycemic controls; Figure 6g), and histological clearance of lipid droplets (Figure 6h). Collectively, these results demonstrate that our eaMRI strategy effectively captures pathological hepatic glucose accumulation in MAFLD—a condition underpinned by insulin resistance‐mediated reduction in glucose disposal rate (e.g., eGDR)—as demonstrated by progressive signal enhancement in diseased mice (Figure 6a,b), while also enabling non‐invasive visualization of therapeutic response, as evidenced in OCA‐treated models (Figure 6e–h).

The biosafety of CrGOx@Lip was preliminarily assessed. Cytotoxicity assays of CrGOx and CrGOx@Lip on L929 cells revealed no significant growth inhibition at Cr concentrations up to 10 mm (Figure S25). Calcein‐AM/PI staining further confirmed negligible apoptosis in L929 cells following co‐incubation (Figure S26). CrGOx@Lip exhibited a blood circulation half‐life (t_1/2_) of 0.56 h (Figure S27). Biodistribution analysis showed that after intravenous injection, the probe was mainly enriched in the liver and spleen, while the presence of chromium in the kidneys was also detected (Figure S28), indicating that the probe and the formed chromium complex are gradually excreted via hepatobiliary and renal routes. Histological sections of major organs (heart, liver, spleen, lungs, kidneys) from mice administered CrGOx@Lip via tail vein injection were examined. H&E staining (Figure S29) showed no evidence of histological alterations or cellular necrosis in these organs at 1 day and 7 days post‐injection, respectively.

## 3 Conclusions

This study was motivated by the urgent need for non‐invasive, specific, and sensitive imaging of glucose metabolism in vivo to advance the understanding and management of cancers and metabolic disorders. Addressing this challenge, we developed an enzyme‐activated MRI (eaMRI) strategy and the corresponding probe, CrGOx, which enables highly sensitive and glucose concentration‐dependent contrast through enzyme‐triggered in situ formation of paramagnetic chromium gluconate.

The probe operates via a cascaded activation mechanism. Specifically, GOx‐catalyzed oxidation of glucose yields gluconate and releases H^+^, which dissolves Cr(OH)_3_ to form chromium gluconate, increasing the water coordination number (*q*) of chromium(III) and resulting in a pronounced *T*_1_ contrast enhancement, exhibiting an 8.28‐fold increase in *r*_1_ relaxivity. To ensure translational applicability, we implemented liposomal encapsulation (CrGOx@Lip), balancing systemic stability with lesion‐specific activation. This design enabled reliable imaging of glucose flux in two distinct pathophysiological settings including glycolytic tumors and MAFLD. In tumor models, CrGOx@Lip visualized the Warburg effect and responded to GLUT1 inhibition, highlighting its utility in cancer diagnostics and therapy monitoring. In MAFLD, it non‐invasively quantified hepatic glucose accumulation and tracked metabolic improvement after obeticholic acid treatment, offering a promising alternative to invasive biopsy.

While ^18^F‑FDG PET remains the clinical gold standard for glucose metabolic imaging, our eaMRI strategy offers two complementary advantages: radiation‑free operation for safe longitudinal monitoring, and direct molecular recognition of glucose itself via enzymatic specificity. Traditional structural and functional MRI techniques offer limited molecular specificity for imaging small metabolic molecules [18, 19]. While magnetic resonance spectroscopy detects metabolic signatures via distinct spectral peaks, its clinical use remains challenging due to reproducibility issues at clinical field strengths [21, 22]. Building upon the pioneering work of glucoCEST MRI in glucose‐dependent contrast [25], our approach offers enhanced specificity by minimizing interference from endogenous metabolites, enabling glucose‑selective signal amplification.

In our design, GOx creates an intimate enzyme–matrix interface that localizes H^+^ production at the nanoparticle surface, enabling efficient Cr(OH)_3_ dissolution and in situ chromium gluconate formation. This confinement minimizes diffusion losses and enhances catalytic turnover for MRI signal amplification. GOx's inherent substrate specificity ensures glucose‐exclusive activation, directly translating to imaging specificity, as confirmed by negligible responses to interferents and clear differentiation in both glycolytic tumors and metabolic liver disease models. By coupling enzymatic recognition with nanomaterial‐enabled amplification, our platform achieves the requisite specificity and sensitivity for molecular imaging.

Some limitations of CrGOx@Lip should be noted. Its hydrodynamic size (∼173 nm), while aiding circulation, may limit penetration into fibrotic or poorly perfused tissues. Additionally, the probe does not resolve cell‐type‐specific glucose uptake within heterogeneous environments. Future designs could employ smaller, activatable probes or tissue‐penetrating peptides to enhance diffusion. Incorporating cell‐specific targeting motifs or multiplexed imaging may help delineate glucose uptake by distinct cellular populations. The probe's activation kinetics are currently limited by partial GOx inactivation and signal transduction. Ongoing work aims to optimize biomineralization conditions to better preserve enzyme activity and enable faster glucose response.

The significant glucose‐dependent *T*_1_ signal amplification achieved through eaMRI demonstrates its promise as a molecular imaging strategy and highlights the potential of trivalent chromium—a biologically essential yet underexplored metal—for bioresponsive MRI. Beyond glucose detection, eaMRI can be adapted for other metabolites by replacing GOx with appropriate oxidases (e.g., lactate oxidase, uricase, glutamate oxidase), and for other signal transducers by substituting Cr^3+^ with alternative paramagnetic ions (e.g., Mn^2^^+^, Fe^3+^, Cu^2^^+^). This modularity positions eaMRI as a versatile molecular imaging platform.

## 4 Materials and Methods

### 4.1 Materials

Cr(NO_3_)_3_•9H_2_O, NaOH, Gox, BSA, cholesterol, soya lecithin, DSPE‐PEG2000, gluconic acid, obeticholic acid, lactose, fructose, galactose, glutamine, glutamic acid, HSA, hemoglobin, and EDTA were purchased from Aladdin Industrial Corporation. BAY‐876 was obtained from Sigma‐Aldrich. Dichloromethane, H_2_O_2_ solution, and HCl solution were provided by Sinopharm Chemical Reagent Co., Ltd. DMEM, paraformaldehyde (4%), and PBS buffer were provided by Biosharp. Glucose assay kit and GOx activity assay kit were purchased from Shanghai Yuanye Co., Ltd. Ultrapure water was used throughout the experiments. All reagents were used without further purification.

### 4.2 Synthesis of CrGOx and CrGOx@Lip

CrGOx and CrBSA were synthesized via a biomimetic mineralization approach using GOx or BSA as a template. Briefly, dissolve 20 mg of Cr(NO_3_)_3_•9H_2_O and 15 mg of GOx in 8 mL of ultrapure water and stir thoroughly for 10 min to obtain a clear solution. Subsequently, slowly add 400 µL of NaOH aqueous solution (1 m) dropwise at 37°C. Continue stirring for 60 min to obtain a pale green, clear solution. Transfer this solution to a dialysis bag (molecular weight cut‐off of 14 000 Da) and dialyze for 6 h. Centrifuge the dialyzed solution (3000 rpm, 10 min) and collect the supernatant to obtain the aqueous solution of CrGOx. The preparation process for CrBSA is identical to that of CrGOx, with BSA replacing GOx in the starting materials.

Dissolve 5 mg of cholesterol, 17 mg of soya lecithin, and 3.7 mg of DSPE‐PEG2000 in 1 mL of dichloromethane. Subsequently, add this solution to 3 mL of the CrGOx aqueous solution and disperse the mixture via probe sonication for 5 min. Remove the dichloromethane using a rotary evaporator to obtain the aqueous solution of CrGOx@Lip. The preparation process for CrBSA@Lip is identical to that of CrGOx@Lip, with CrBSA replacing CrGOx in the starting materials.

### 4.3 Characterizations

The morphology of CrGOx and other relevant nanomaterials was examined by transmission electron microscopy using an FEI Tecnai G2 F20 microscope. Elemental mapping, and energy‐dispersive spectroscopy of CrGOx were performed on an FEI F200 microscope. The hydrodynamic diameter distribution and zeta potential of CrGOx and related nanomaterials in aqueous solution were measured by dynamic light scattering (Malvern Nano ZS100). UV–vis absorption spectra for all materials were recorded using an Agilent Cary 60 spectrophotometer. The Cr concentration in CrGOx was quantitatively determined by inductively coupled plasma mass spectrometry (Agilent 720ES). X‐ray photoelectron spectroscopy (PHI 5000C) was employed to analyze the elemental composition and the chemical valence state of Cr in CrGOx. The crystal structure of CrGOx was characterized by X‐ray diffraction (Rigaku D). The chemical state and coordination environment of Cr in CrGOx were investigated using X‐ray absorption fine structure spectroscopy (SSRF‐ BL11B); the acquired XAFS data were analyzed using the Athena and Artemis software, with data visualization performed in Origin. The secondary structure of free GOx and CrGOx was analyzed by circular dichroism spectroscopy using a JASCO FVS‐6000 spectropolarimeter.

### 4.4 GOx Activity of CrGOx

The GOx activity of CrGOx was assessed by using a commercial GOx activity assay kit. Add 100 µL of free GOx or CrGOx solution (GOx 1 mg/mL) to 900 µL of the assay kit working solution. Immediately transfer the mixture to a UV–vis spectrophotometer and record the absorption spectrum after 10 s. Subsequently, incubate the reaction mixtures at 37°C and record their absorption spectra again after 60 min. Calculate the difference in absorbance (ΔA) at 500 nm between the final and initial measurements for each sample. The relative activity of GOx within CrGOx compared to free GOx is determined by the ratio ΔA(CrGOx)/ΔA(free GOx).

### 4.5 Glucose‐responsive Relaxation Study of CrGOx

The *T*_1_ and *T*_2_ relaxation time of CrGOx across a glucose concentration range (0–10 mm) were measured by using a Bruker minispec mq 60 MR analyzer (1.41 T). The *r*_1_ and *r*_2_ relaxivities were determined from the slopes of the linear regressions of 1/*T*_1_ (s^−1^) and 1/*T*_2_ (s^−1^) vs. Cr concentration (mm), analyzed using GraphPad Prism software.

For kinetic study, the *T*_1_ relaxation times of CrGOx were monitored over time at varying glucose concentrations using the MR analyzer. Specifically, 500 µL of glucose aqueous solution was added to 500 µL of CrGOx (4 mm Cr) to achieve final glucose concentrations of 0, 0.5, 1, 2.5, 5, and 10 mm. *T*_1_ relaxation times were measured at 0, 30, 60, 90, 120, 150, and 180 min.

A NIUMAG MRI scanner (1.0 T) was used to record the in vitro *T*_1_‐weighted MR images of CrGOx. Glucose aqueous solutions were added to CrGOx aqueous solutions to achieve final Cr concentrations of 0.13, 0.25, 0.5, 1, and 2 mm, and final glucose concentrations of 0, 0.5, 1, 2.5, 5, and 10 mm. The reaction mixtures were incubated at 37°C for 6 h, followed by MR scanning. Scanning sequence: TR/TE = 360 ms/18.14 ms, FOV Read = 60 mm, FOV Phase = 60 mm, slice count = 5, slice thickness = 2 mm, slice gap = 0.5 mm, echo position = 50%, read size = 256, phase size = 192. The NIUMAG NMR imaging processing software was used to analyze the MR signal intensity of each sample.

To evaluate the glucose response specificity of CrGOx, we measured the *T*_1_ relaxation times and MRI signal intensities of CrGOx under various physiological conditions and interferents. Briefly, 500 µL of 10 mm aqueous solutions of glucose, lactose, fructose, galactose, glutamine, glutamic acid, HSA, or hemoglobin was added to 500 µL of 3 mm CrGOx aqueous solution. CrGOx in a pH 6.5 buffer was also prepared. After 6 h of incubation, relaxation time measurements and MR imaging were performed.

### 4.6 Glucose‐Responsive Mechanism Study of CrGOx

First, the relaxation response of CrGOx to H_2_O_2_ and gluconic acid was evaluated. Aqueous solutions of CrGOx containing 0, 0.25, 0.5, 2.5, 5, or 10 mm H_2_O_2_ were prepared, and their *T*_1_ relaxation times were measured over 180 min using the MR analyzer. Similarly, CrGOx solutions containing 0, 0.25, 0.5, 1.25, 2.5, or 5 mm gluconic acid were prepared, with *T*_1_ relaxation times monitored for 180 min. Additionally, CrGOx and CrBSA samples containing 0–5 mm gluconic acid were prepared and subjected to MR imaging after 6‐hr incubation. The responsiveness of CrGOx and CrBSA to gluconate and hydrogen ions was also tested. Sodium gluconate (pH 7.4) is prepared with gluconic acid and 1 mm sodium hydroxide solution. Hydrogen ions are derived from dilution with hydrochloric acid.

For the mechanism study, the products of CrGOx‐glucose reaction were analyzed. 1 mL CrGOx (4 mm Cr) was added to 1 mL glucose (10 mm) solution. After a 6‐h reaction, the mixture was centrifuged at 15 000 rpm for 15 min. The resulting supernatant and precipitate were reconstituted to 2 mL total volume and subjected to UV–vis spectroscopy. MR imaging of CrGOx, CrGOx‐glucose reaction mixture, supernatant, and precipitate was performed using the MRI scanner, with signal intensities quantified by the NIUMAG NMR imaging processing software. In parallel, reaction solutions containing 0–20 mm glucose were centrifuged. The supernatant and precipitate fractions at each glucose concentration were imaged by the MRI scanner, and their signal intensities were quantified. The components of the supernatant were detected by high‐resolution mass spectrometry (Thermo Fisher Scientific Q Exactive Focus).

Cr^3+^ ions released from CrGOx‐glucose reactions were quantified using EDTA chelation. A calibration curve was established by preparing Cr^3+^‐EDTA complexes (Cr^3+^: 0–10 mm) and measuring their UV–vis absorption spectra. Absorbance at 540 nm was plotted against Cr^3+^ concentration to generate the standard curve. For test samples, 1 mL of supernatant from centrifuged reaction mixtures (glucose: 0–20 mm) was mixed with 1 mL of EDTA solution (5 mg/mL). After a 2‐h reaction at 65°C, UV–vis spectra were acquired. Cr^3+^ concentrations in the supernatants were determined from the calibration curve.

### 4.7 Cell Experiments

L929 cells were seeded in 96‐well plates at densities ranging from 5 × 10^3^ to 8 × 10^3^ cells per well and permitted 12 h for attachment. Following this incubation, the culture medium was exchanged for glucose‐free medium supplemented with either CrGOx or CrGOx@Lip at graded chromium concentrations. After 24 h of incubation, cells underwent PBS washing before fresh medium containing CCK‐8 reagent was introduced. Subsequent to a 2 h incubation period, optical density measurements were performed using a microplate reader to quantify cellular viability. Following the standard Calcein‐AM/PI dual‐staining protocol, cells were incubated with the staining solution for 30 min post‐treatment. Subsequent fluorescence microscopy examination enabled visualization of live/dead cell distributions.

### 4.8 Animal Experiments

All animal experiments in this paper were performed according to the guidelines published by the Animal Ethics Committee of Tongji University (approval number: TJAA08125401). We confirm that tumor size did not exceed 10% of the animal's body weight and that tumor volume did not exceed 2000 mm^3^ (or 20 mm in diameter) at any time during the study (Table S2). We confirm that animal body weight loss did not exceed 15% of the original body weight at any point during the study (Table S3).

### 4.9 *T*_1_‐Weighted Tumor MRI

Six‐week‐old BALB/c mice were used to establish CT26 tumor models. A 100 µL suspension of CT26 cells (1 × 10^6^ cells) in PBS was injected subcutaneously into the right hind flank. Tumor volume was monitored daily and calculated as V = (length × width^2^)/2. When tumors reached 300–400 mm^3^, the mice were subjected to imaging studies. All animals were fasted for 12 h prior to experiments.

Tumor‐bearing mice were positioned within the MR scanner for baseline *T*_1_‐weighted imaging. Following imaging, mice were removed and administered an intratumoral injection of 50 µL CrBSA or CrGOx (Cr: 4 mm). Subsequent MR imaging was performed at 10, 20, 40, and 60 min post‐injection. Signal intensity analysis was conducted using the NIUMAG NMR imaging processing software.

Mice were divided into three groups: (1) one group received daily intraperitoneal administration of BAY‐876 (5 mg/kg). After 8 days, these mice were intravenously injected via the tail vein with 200 µL CrGOx@Lip (80 mg/kg); (2) the other two groups consisted of untreated mice received CrGOx@Lip or CrBSA@Lip via tail vein injection, respectively. All three groups were transferred to the MR scanner for imaging. At 120 min post‐probe administration, each mouse received an intraperitoneal injection of 400 µL of 0.1 m glucose, followed by continued MR imaging monitoring. Following imaging, tumors were excised from all mice and subjected to sectioning and GLUT1 immunofluorescence staining.

### 4.10 *T*_1_‐weighted MAFLD MRI

Fourteen‐week‐old MAFLD model mice were obtained from Jicui Biological Co., Ltd. Both healthy control mice and MAFLD mice underwent baseline *T*_1_‐weighted MRI scanning. Following imaging, all mice received an intravenous injection of 200 µL CrGOx@Lip (80 mg/kg) via the tail vein. Subsequent MR imaging was performed at 60, 120, 180, and 240 min post‐injection. Liver signal intensity was quantified using imaging software.

MAFLD mice were divided into three treatment groups receiving oral obeticholic acid (30 mg/kg, administered every other day). The groups received either 1, 3, or 9 doses, respectively. After completing the treatment regimen, all mice were intravenously administered 200 µL CrGOx@Lip (80 mg/kg) and transferred to the MR scanner for *T*_1_‐weighted imaging. Upon completion of imaging, liver tissues were harvested from all mice for sectioning and H&E staining.

Liver tissues from each group were weighed and homogenized in ice‐cold physiological saline at a tissue‐to‐saline ratio of 1:9 (w/v). The homogenates were centrifuged at 3000 rpm for 10 min. Subsequently, 0.02 mL of supernatant was mixed with 2 mL of freshly prepared GOD‐POD working solution (from the assay kit) and incubated at 37°C for 15 min. Absorbance at 505 nm was measured using a UV–vis spectrophotometer. For standard curve generation, serial dilutions of glucose standard solution (provided in the kit) were prepared, and their corresponding absorbance values were measured. A calibration curve was generated through linear regression. Hepatic glucose content was calculated based on the regression equation.

### 4.11 In Vivo MRI Parameters and Statistical Analysis

In vivo MRI scan sequence was set as follows: TR/TE = 360 ms/18.14 ms, FOV Read = 100 mm, FOV Phase = 100 mm, slice count = 7, slice thickness = 2.5 mm, slice gap = 0.5 mm, echo position = 50%, read size = 256, phase size = 192. For each animal, three regions of interest (ROI) with >100 pixels were manually drawn within the tumor to quantify signal intensity.

The SNR and ΔSNR% were calculated using the following Equations:

$$SNR=\frac{SI_{\mathrm{mean}}}{SD_{\mathrm{air}}}$$

$$\Delta SNR\%=\frac{\left(\mathrm{SN}R_{\mathrm{after}}-\mathrm{SN}R_{\mathrm{before}}\right)}{\mathrm{SN}R_{\mathrm{before}}}\times 100\%$$

SI_mean_ denotes the mean intensity of ROI. SD_air_ signifies the standard deviation of the signal in the air outside the animal.

### 4.12 In Vivo Safety Assessment

Healthy BALB/c mice were intravenously injected with CrGOx@Lip (80 mg/kg) via the tail vein. At predetermined time points (0.5, 1, 2, 3, 4, 8, 12, and 24 h post‐injection), blood samples were collected from the retro‐orbital plexus into heparin‐coated tubes. The chromium concentration in each blood sample was quantified by ICP‐MS after digestion with aqua regia.

At 0, 0.5, 1, 4, 8, 12 and 24 h post‐intravenous injection of CrGOx@Lip (80 mg/kg), mice were humanely euthanized, and major organs (heart, liver, spleen, lungs, and kidneys) were surgically harvested. Tissue samples were weighed, digested in aqua regia, and diluted with ultrapure water. Chromium content in each organ was determined by ICP‐MS.

To assess the in vivo biocompatibility, healthy BALB/c mice were intravenously injected with CrGOx@Lip (80 mg/kg) and euthanized at 1 day and 7 days post‑administration. Major organs were immediately harvested, fixed in 4% paraformaldehyde, embedded in paraffin, and sectioned into slices. Tissue sections were stained and examined under a light microscope for histopathological evaluation. Untreated mice served as controls.

Statistical analyses were conducted using GraphPad Prism 9.0 software and Origin 2021. Data are presented as mean ± SD. Data comparisons were made using one‐way or two‐way analysis of variance (ANOVA).

## Author Contributions

Y.X. and W.Y. contributed equally to this work. Y.X. and B.Z. co‐designed the project and experiments. Y.X., W.Y., Y.Y., H.W., Z.W., and Y.Y. performed all the experiments. Y.X. and B.Z. analyzed the data and wrote the manuscript. All authors discussed the results and reviewed the manuscript.

## Conflicts of Interest

The authors declare no conflicts of interest.

## Supporting information




**Supporting File**: advs74861‐sup‐0001‐SuppMat.docx.

## Data Availability

The data that support the findings of this study are available from the corresponding author upon reasonable request.
