# Supplementary material for: Enzyme‐Activated MRI for In Vivo Glucose Imaging via a Biodegradable Chromium Nanoprobe
Source: Adv Sci (Weinh). 2026 Mar 15;13(30):e74861. doi: 10.1002/advs.74861 (PMC13248796; doi:10.1002/advs.74861)
Supplement: Supplementary file 1 — Supporting File: advs74861‐sup‐0001‐SuppMat.docx. [file ADVS-13-e74861-s001.docx]

Supplementary Materials for

**Enzyme-Activated MRI for In Vivo Glucose Imaging via a Biodegradable Chromium Nanoprobe**

Yan Xu*^1,2#^*, Weitao Yang*^1#^*, Yanjing Yun*^1^*, Hui Wang*^1^*, Zhuoyao Wu*^1^*, Youyi Yu*^1^*, and Bingbo Zhang*^1^**

*^1^*Department of Radiology, Tongji Hospital, Shanghai Frontiers Science Center of Nanocatalytic Medicine, the Institute for Biomedical Engineering & Nano Science, School of Medicine, Tongji University, Shanghai 200065, China.

*^2^*Shanghai Key Laboratory of Molecular Imaging, Shanghai Pudong New Area Gongli Hospital Affiliated Shanghai University of Medicine and Health Sciences, Shanghai, 201318, China.

*Corresponding author. Email: bingbozhang@tongji.edu.cn


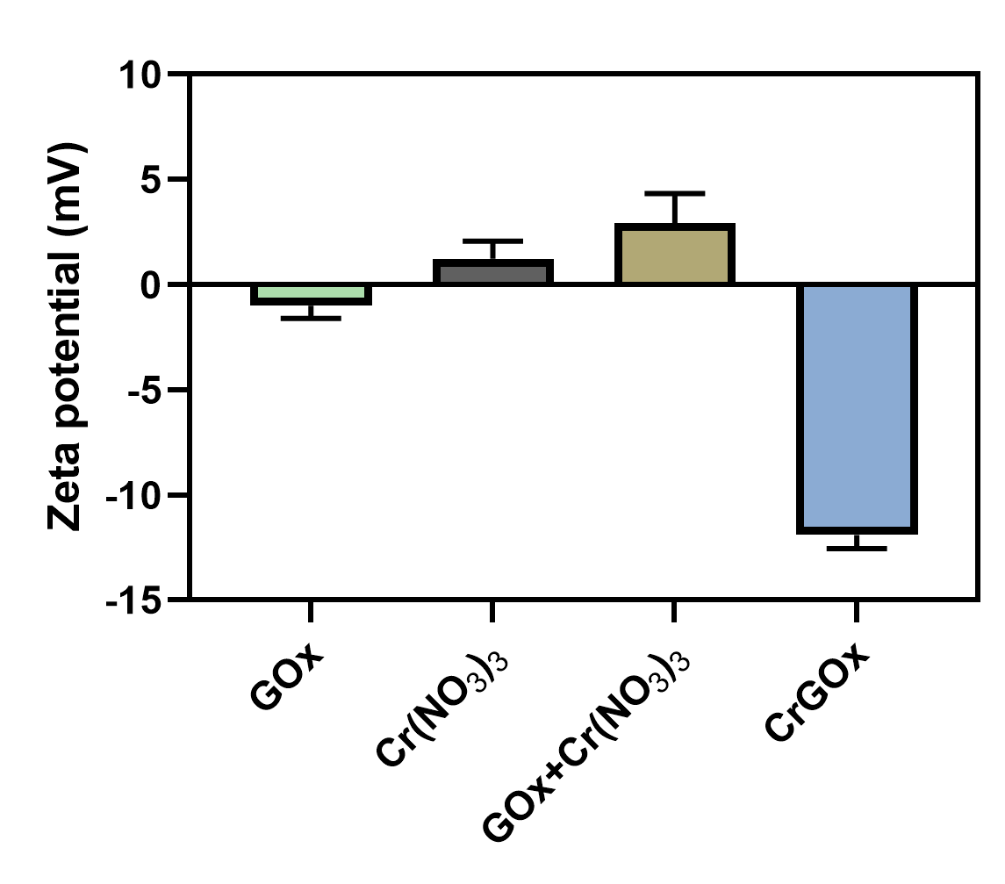


**Figure S1.** Zeta-potential of GOx, Cr(NO_3_)_3_, GOx+Cr(NO_3_)_3_, and CrGOx in distilled water.


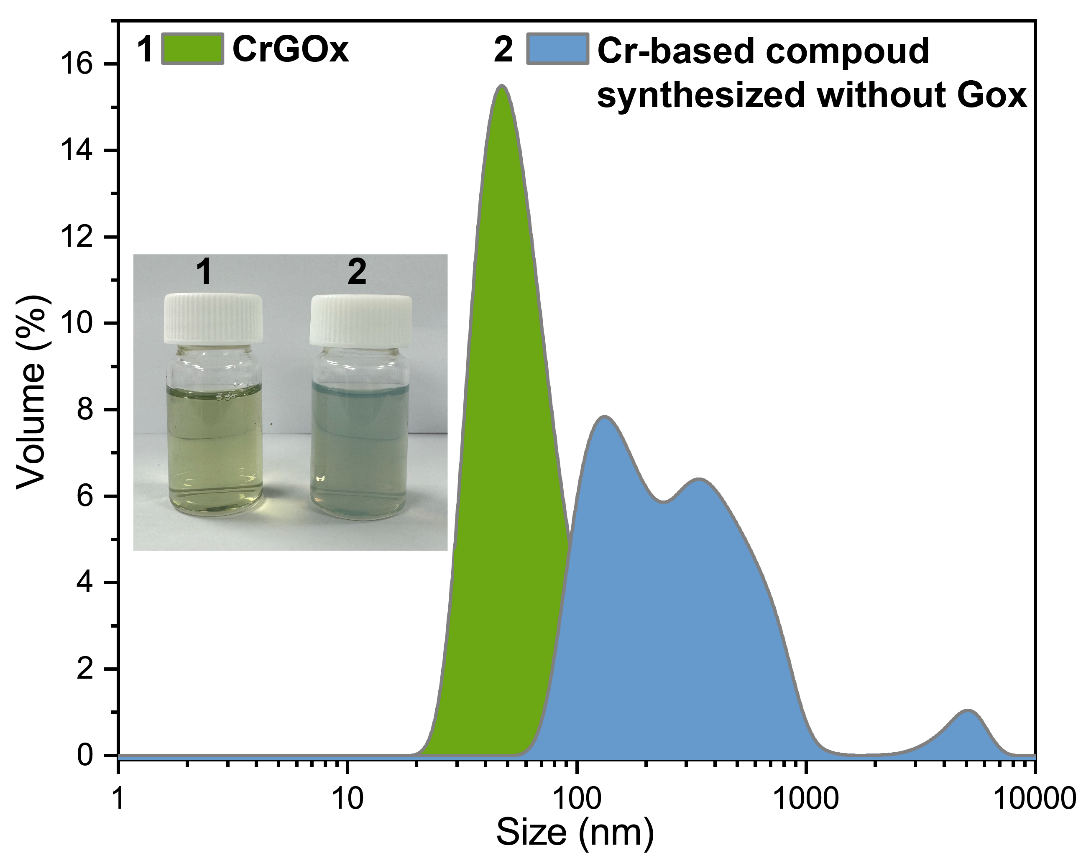


**Figure S2.** Hydrodynamic size and inserted digital pictures of CrGOx, and Cr-based compoud synthesized without GOx.


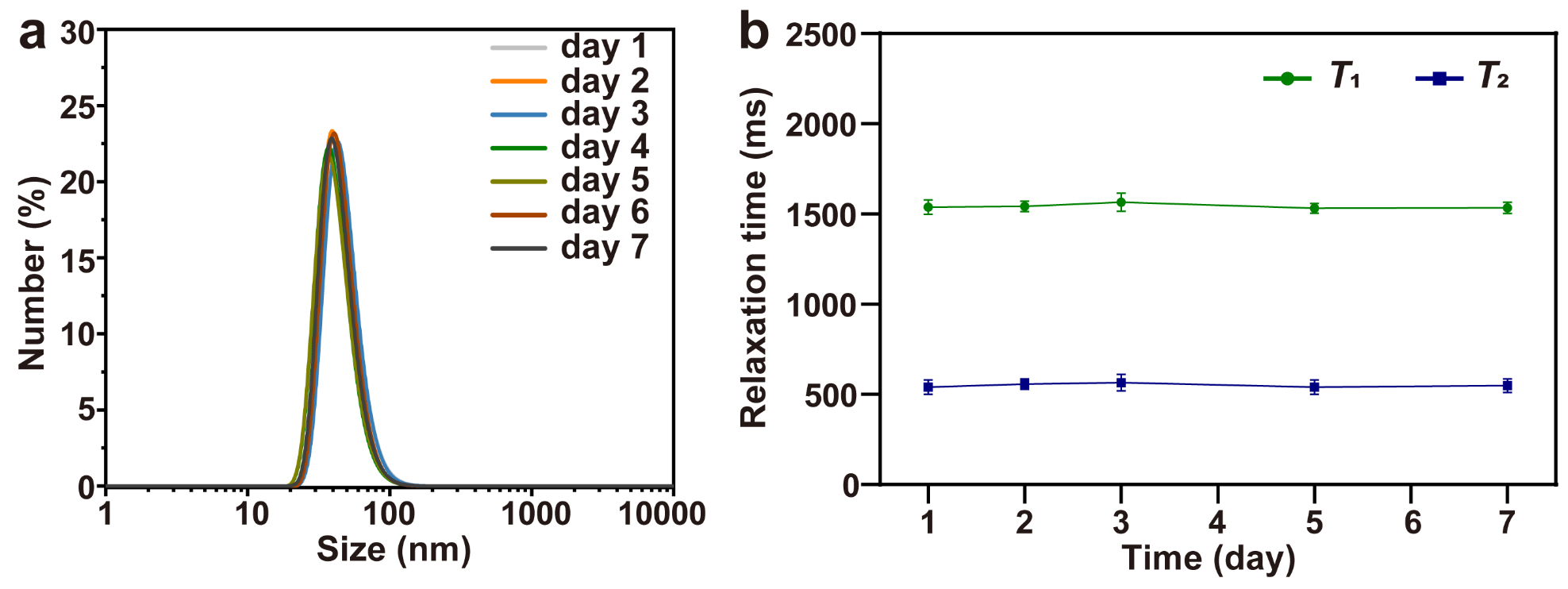


**Figure S3.** (a) Hydrodynamic size of CrGOx in fetal bovine serum over 7 days. (b) *T*_1_ and *T*_2_ relaxation times of CrGOx measured over 7 days.


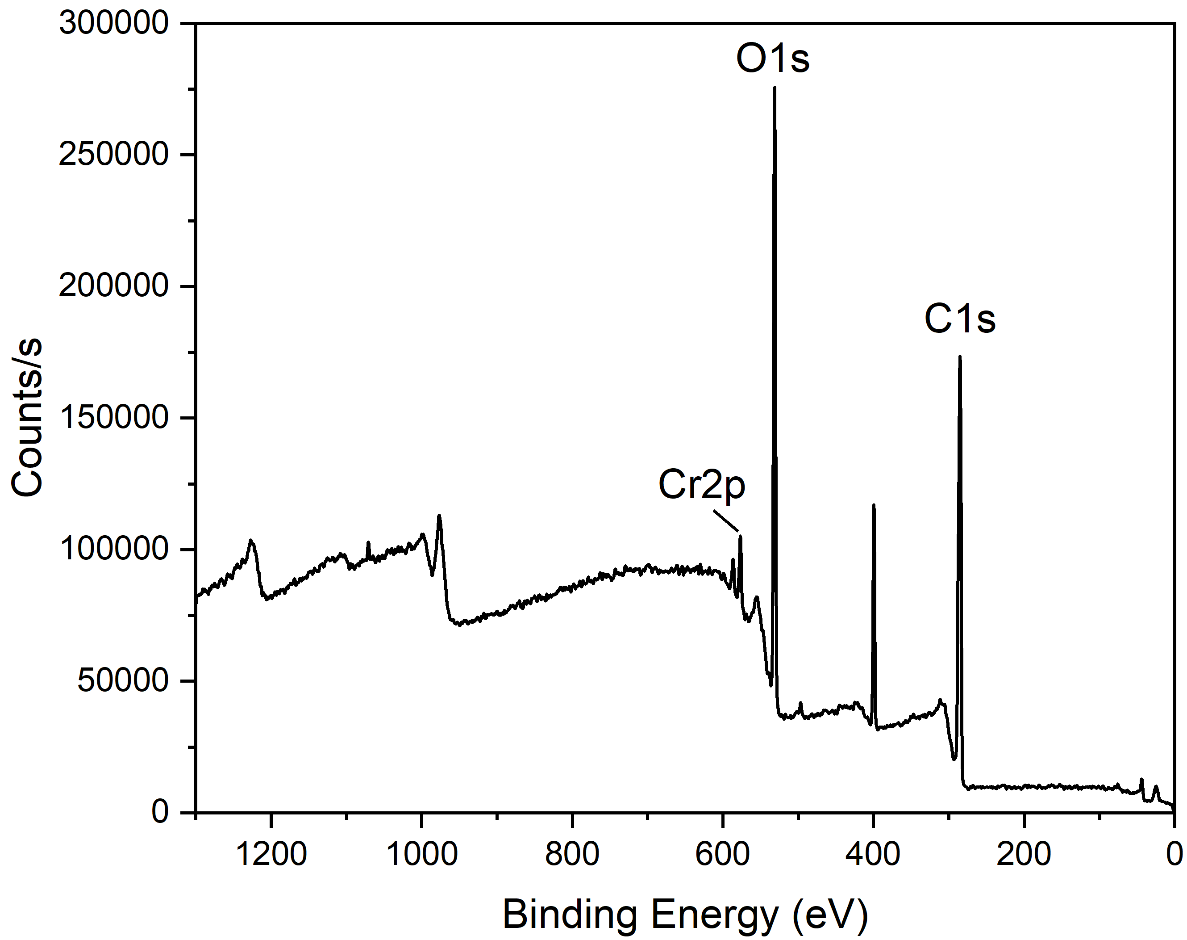


**Figure S4.** XPS compositional analysis of CrGOx.


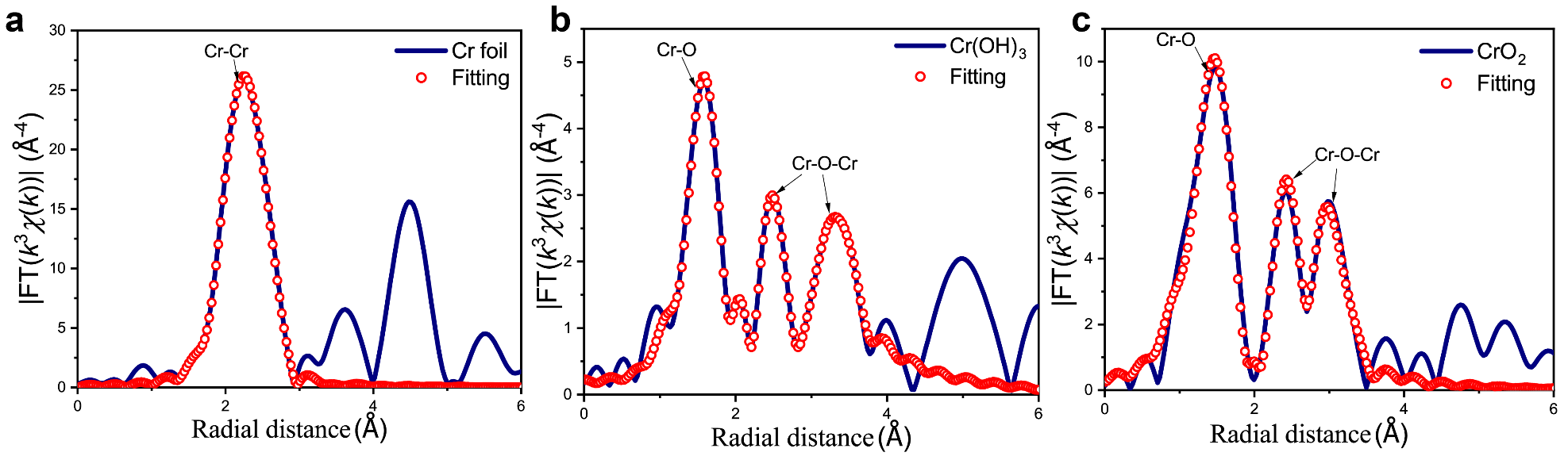


**Figure S5.** Fitting curves of EXAFS spectra for (a) Cr foil, (b) Cr(OH)_3_, and (c) CrO_2_ at R-space.


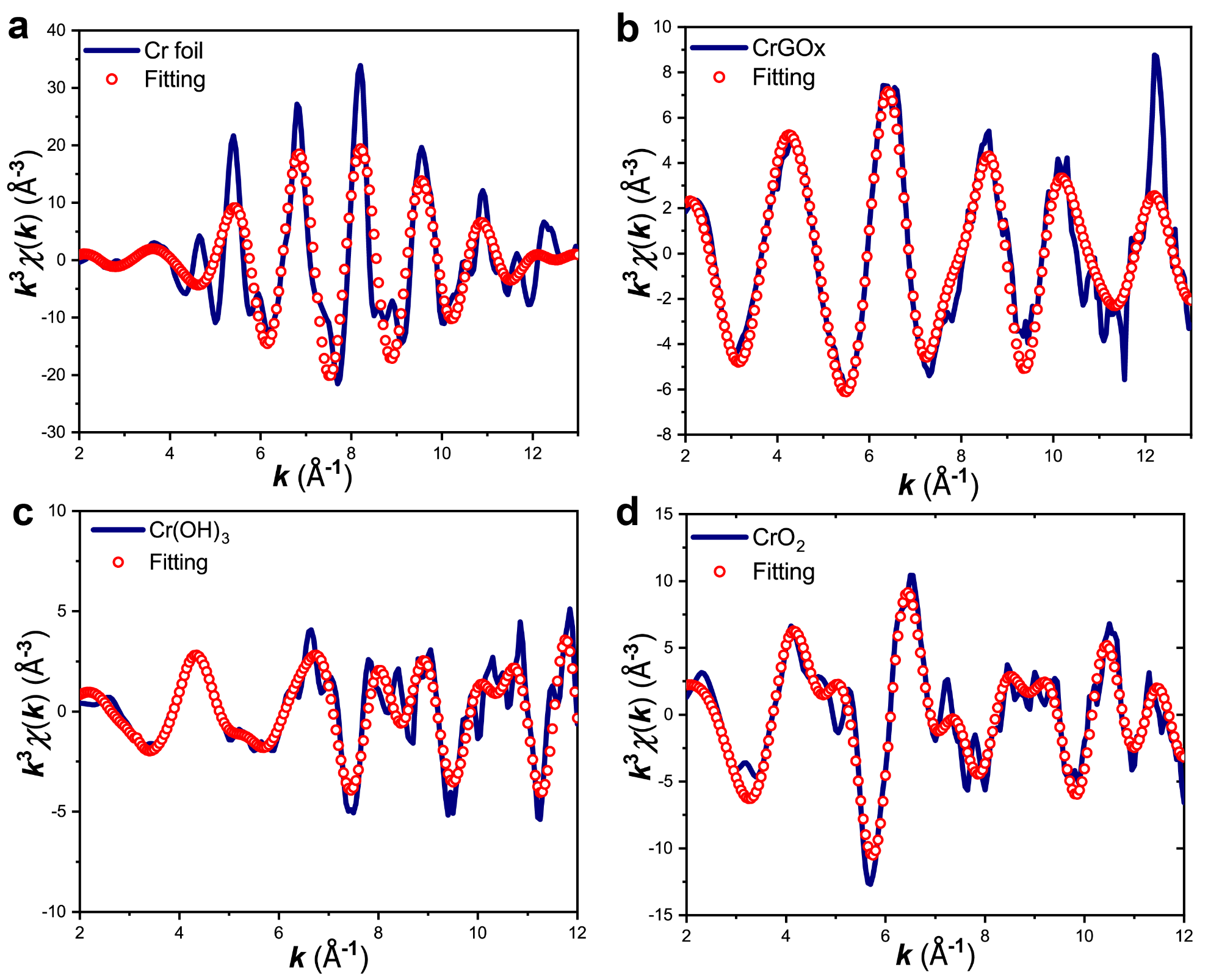


**Figure S6.** Fitting curves of EXAFS spectra for (a) Cr foil, (b) CrGOx, (c) Cr(OH)_3_, and (d) CrO_2_ at k-space.


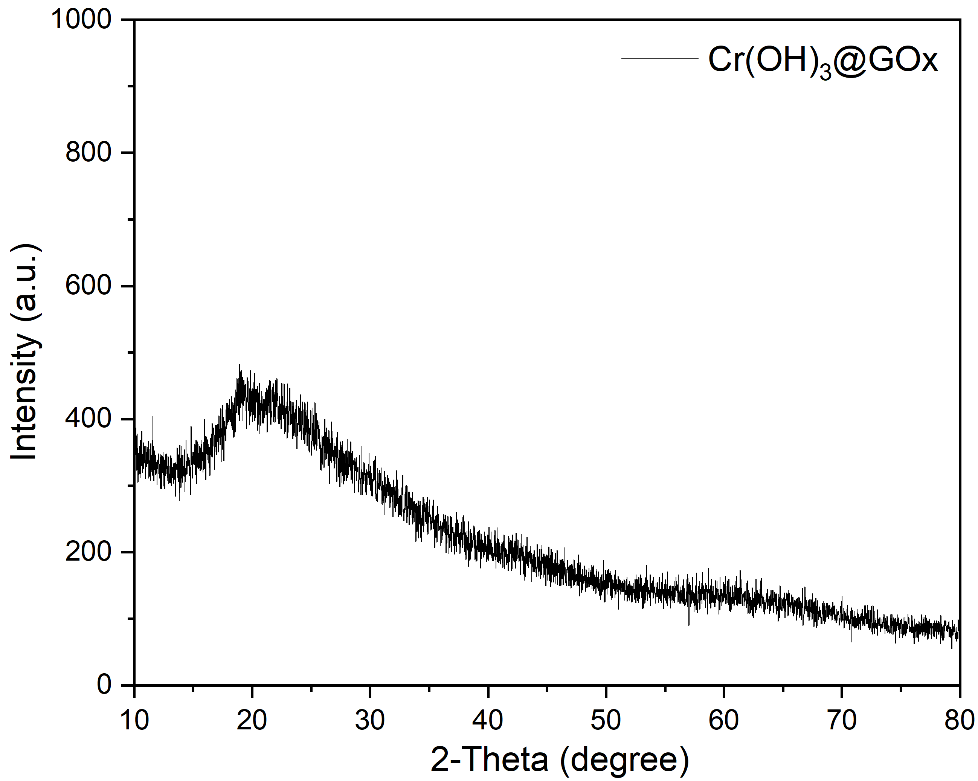


**Figure S7.** XRD spectrum of CrGOx.





**Figure S8.** SAED pattern of CrGOx.


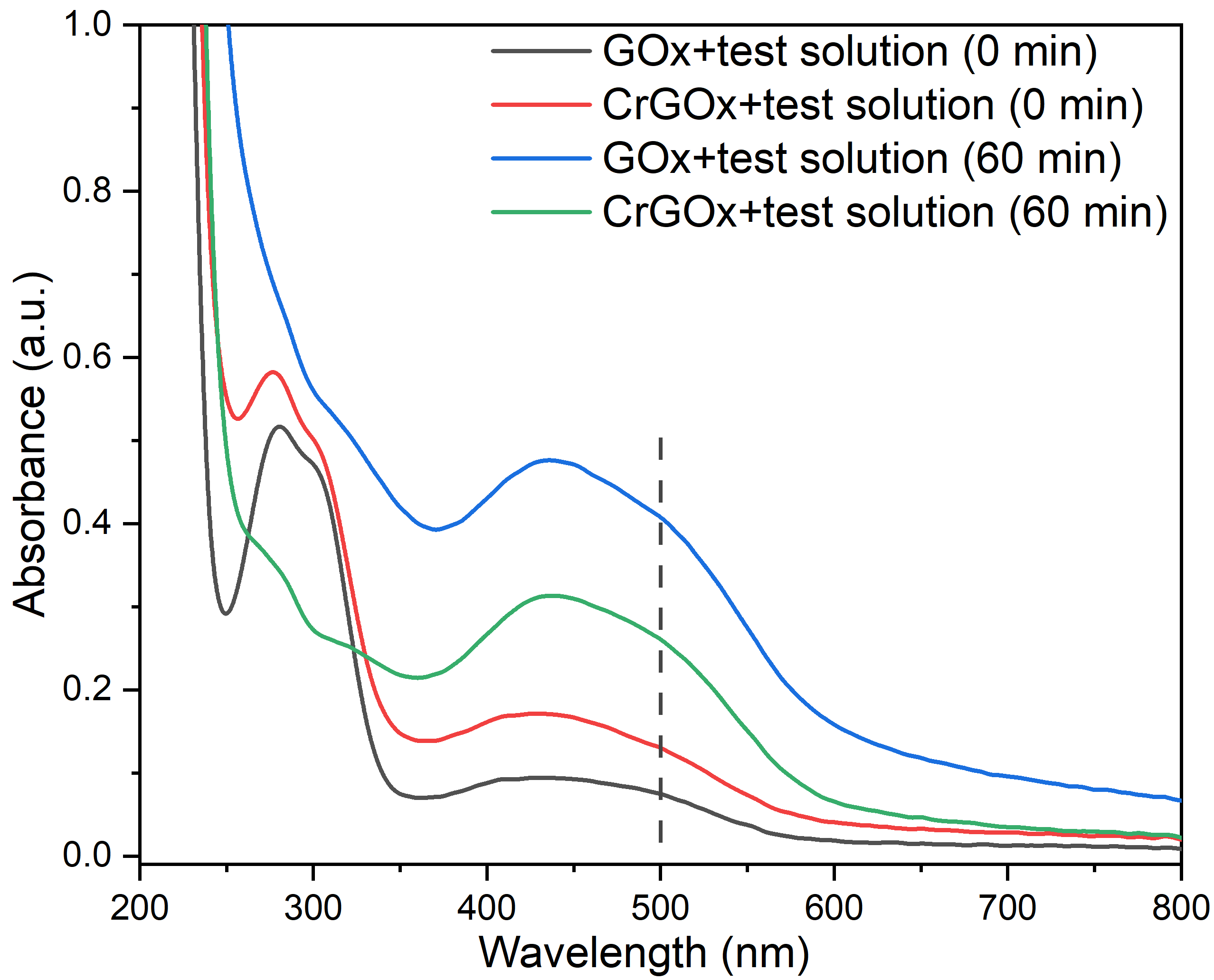


**Figure S9.** The UV-Vis absorption spectra of free GOx and CrGOx tested by GOx activity assay kit.


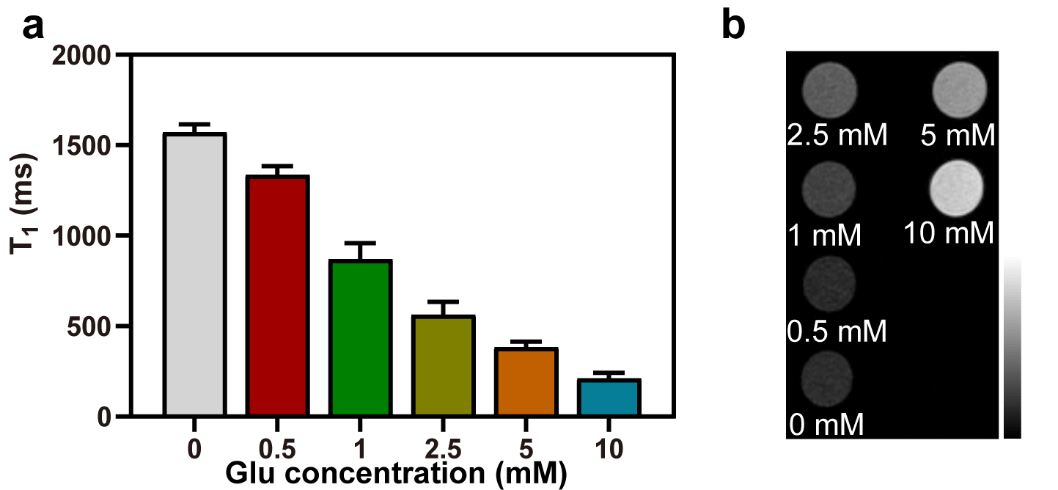


**Figure S10.** (a) *T*_1_ relaxation time and (b) *T*_1_-weighted imaging pictures of CrGOx in fetal bovine serum with varying glucose concentrations.


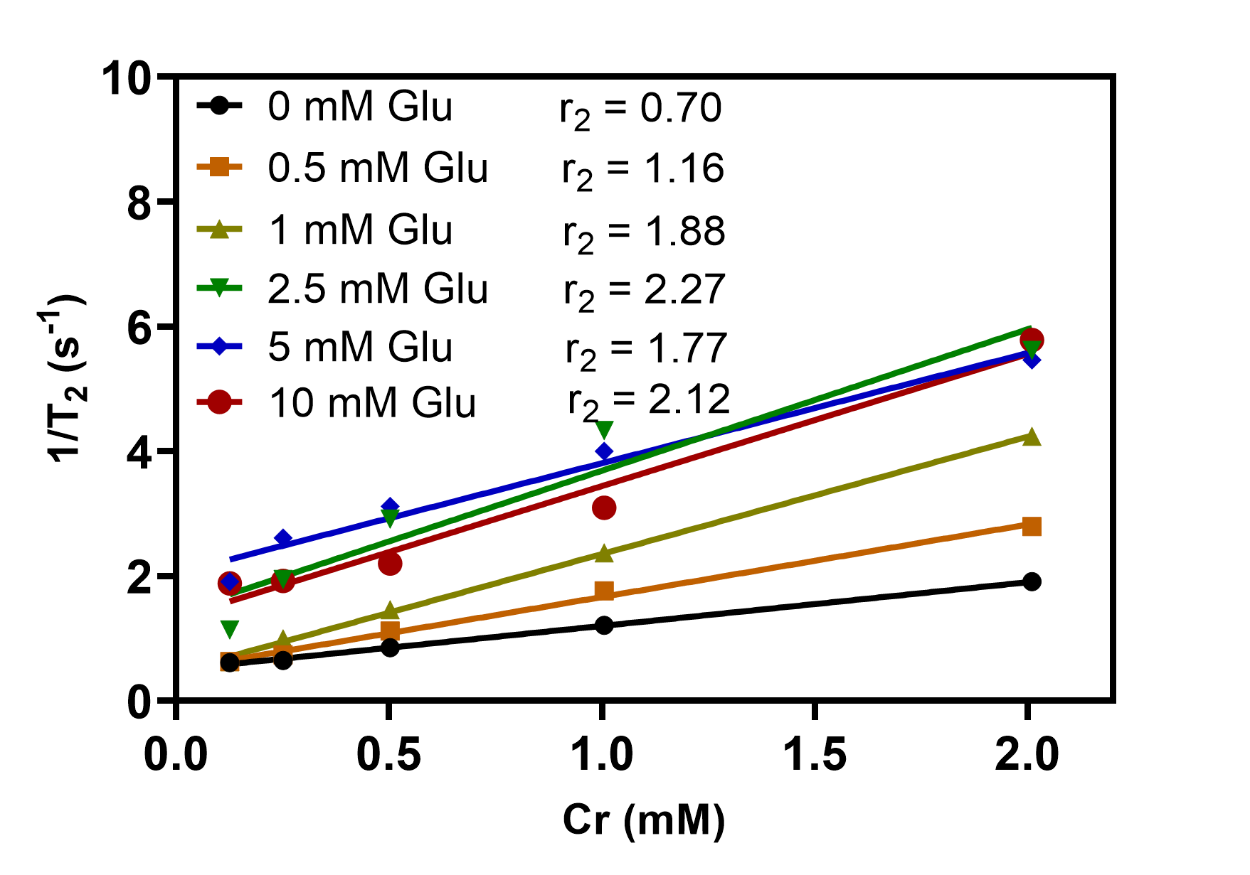


**Figure S11.**The transverse relaxation curve fitting of CrGOx under different glucose concentration.


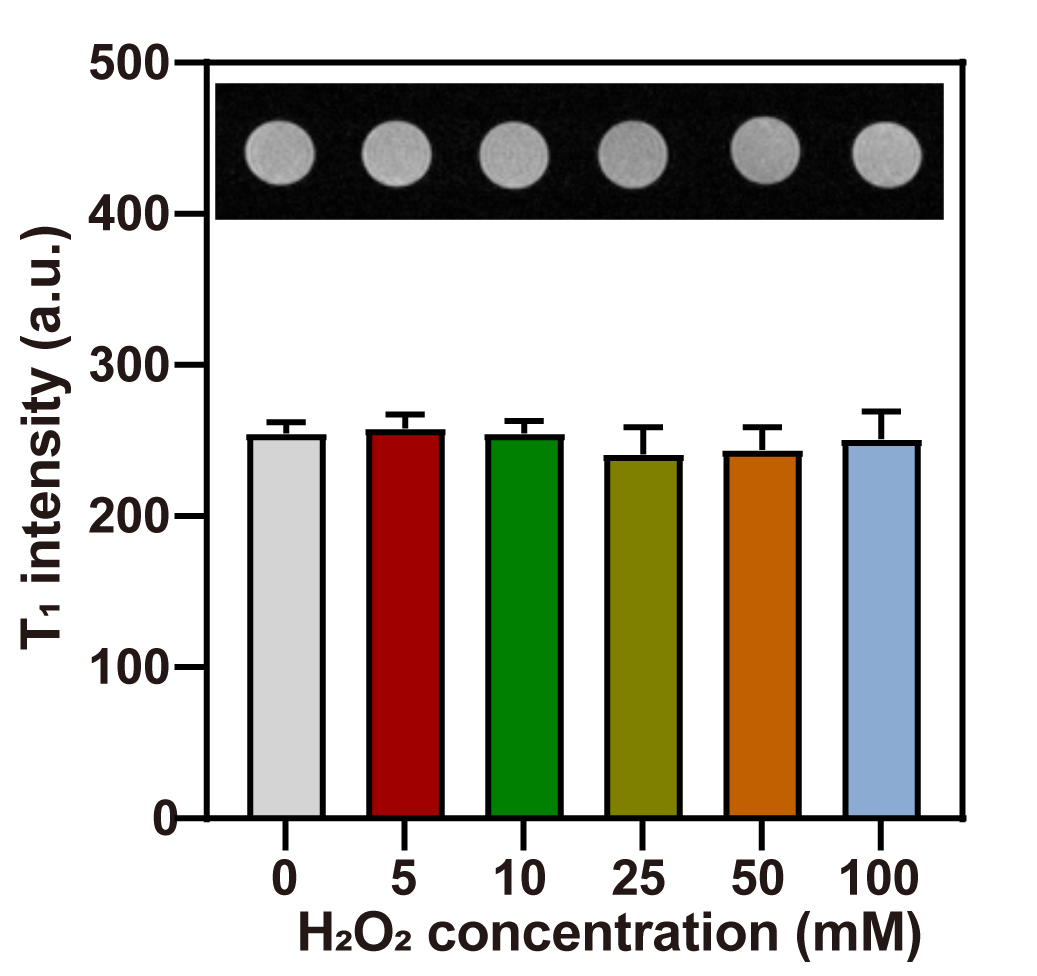


**Figure S12.** *T*_1_-weighted imaging pictures and quantitative signal intensity of Cr^3+^ ions incubated with H_2_O_2_ (0-100 mM) for 3 hours.


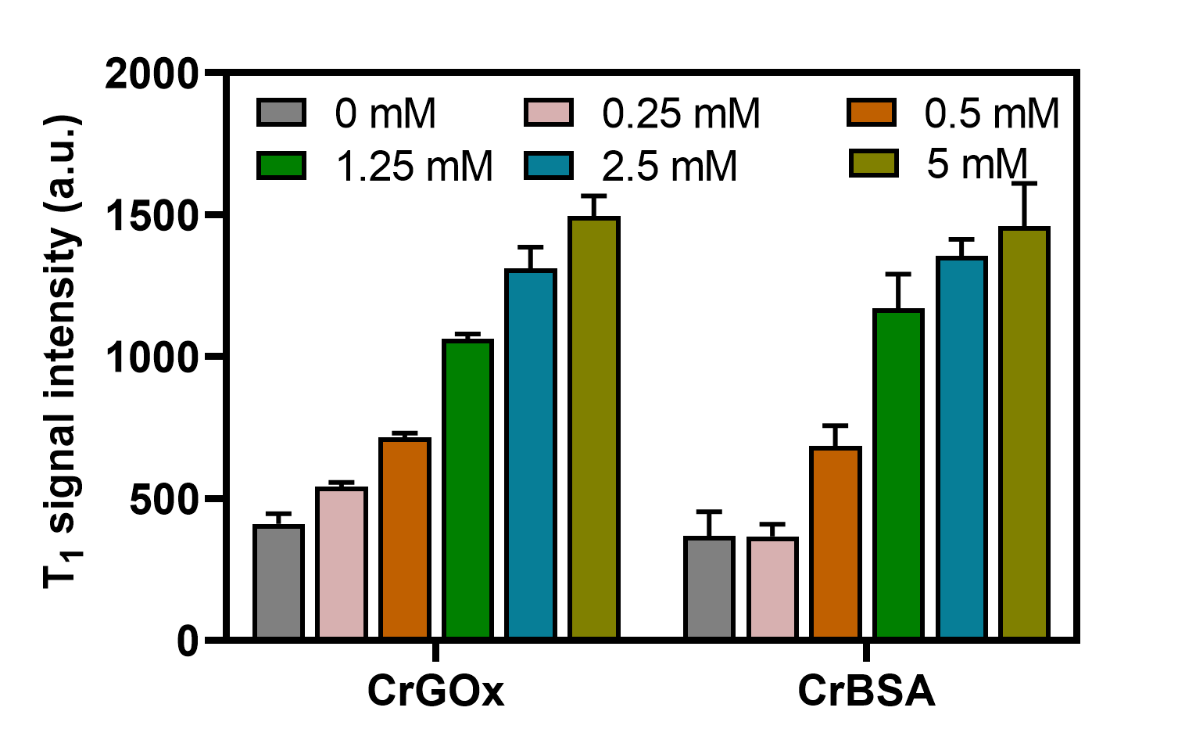


**Figure S13.** *T*_1_-weighted signal intensity of CrGOx and CrBSA under various gluconic acid concentration.


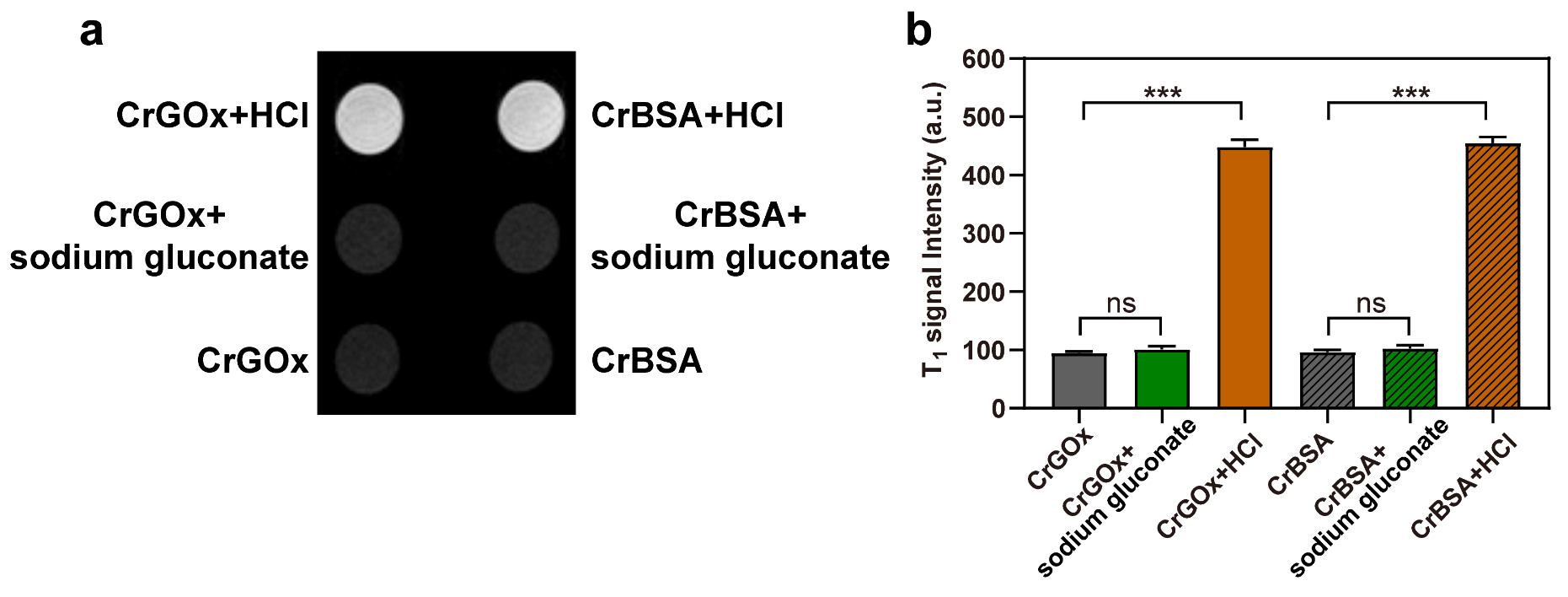


**Figure S14.** (a) *T*_1_-weighted MRI, and (b) signal intensity of CrGOx, CrGOx incubated with sodium gluconate (10 mM, pH 7.4 adjusted with NaOH)—CrGOx+sodium gluconate, CrGOx incubated with HCl (10 mM)—CrGOx+HCl, CrBSA, CrBSA +sodium gluconate, and CrBSA+HCl.


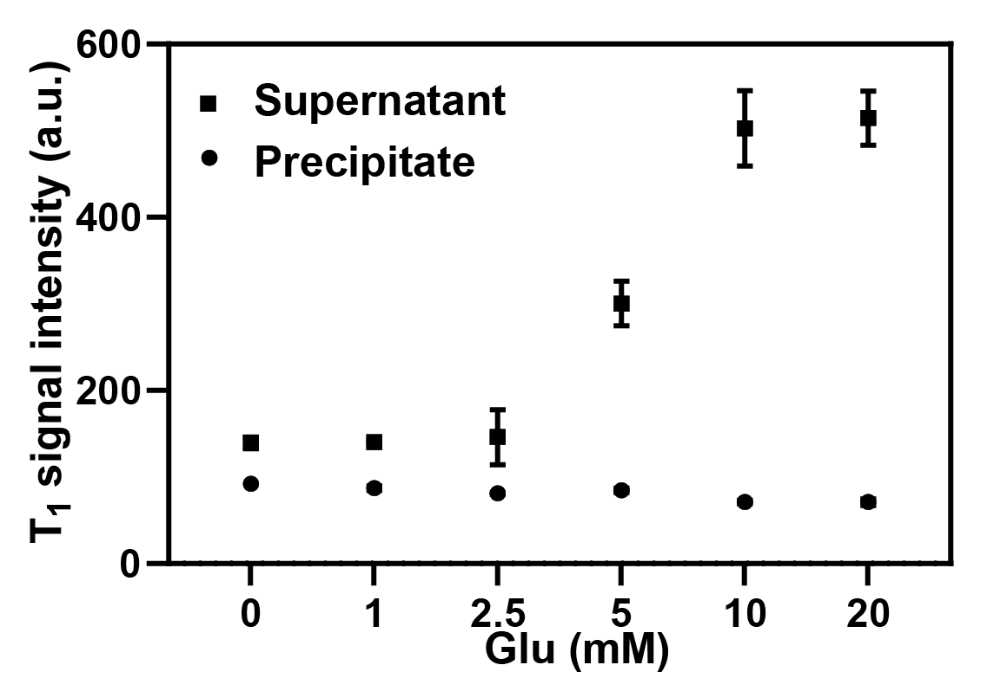


**Figure S15.** In vitro *T*_1_ MRI signal intensity of the supernatant, and the precipitate across varying glucose concentrations.


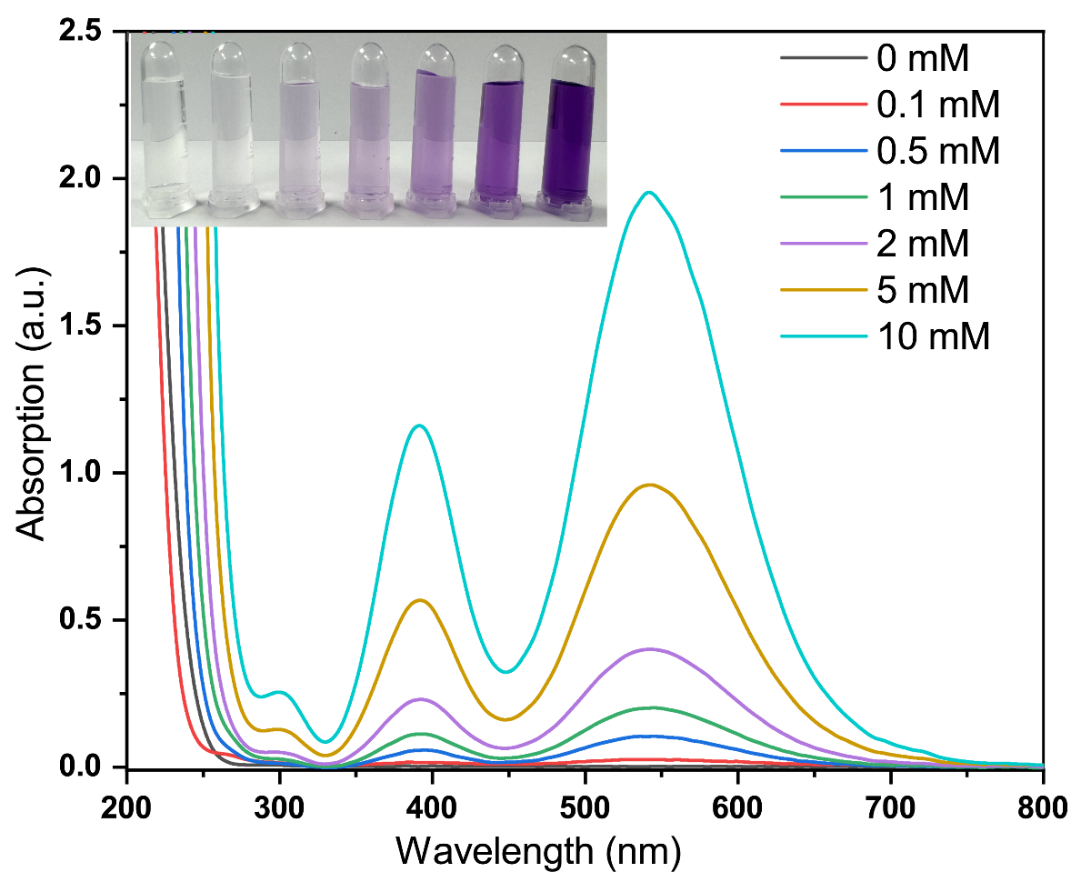


**Figure S16.** UV-Vis absorption spectra and an inserted picuture of EDTA with gradient Cr^3+^ concentrations (0-10 mM).


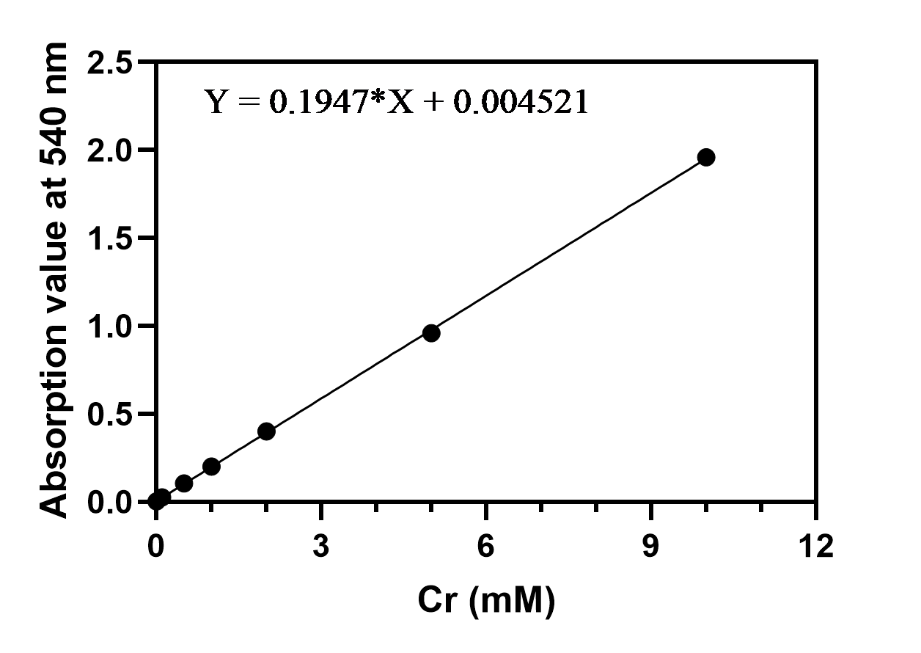


**Figure S17.** The calculated standard curve of Cr^3+^-EDTA.


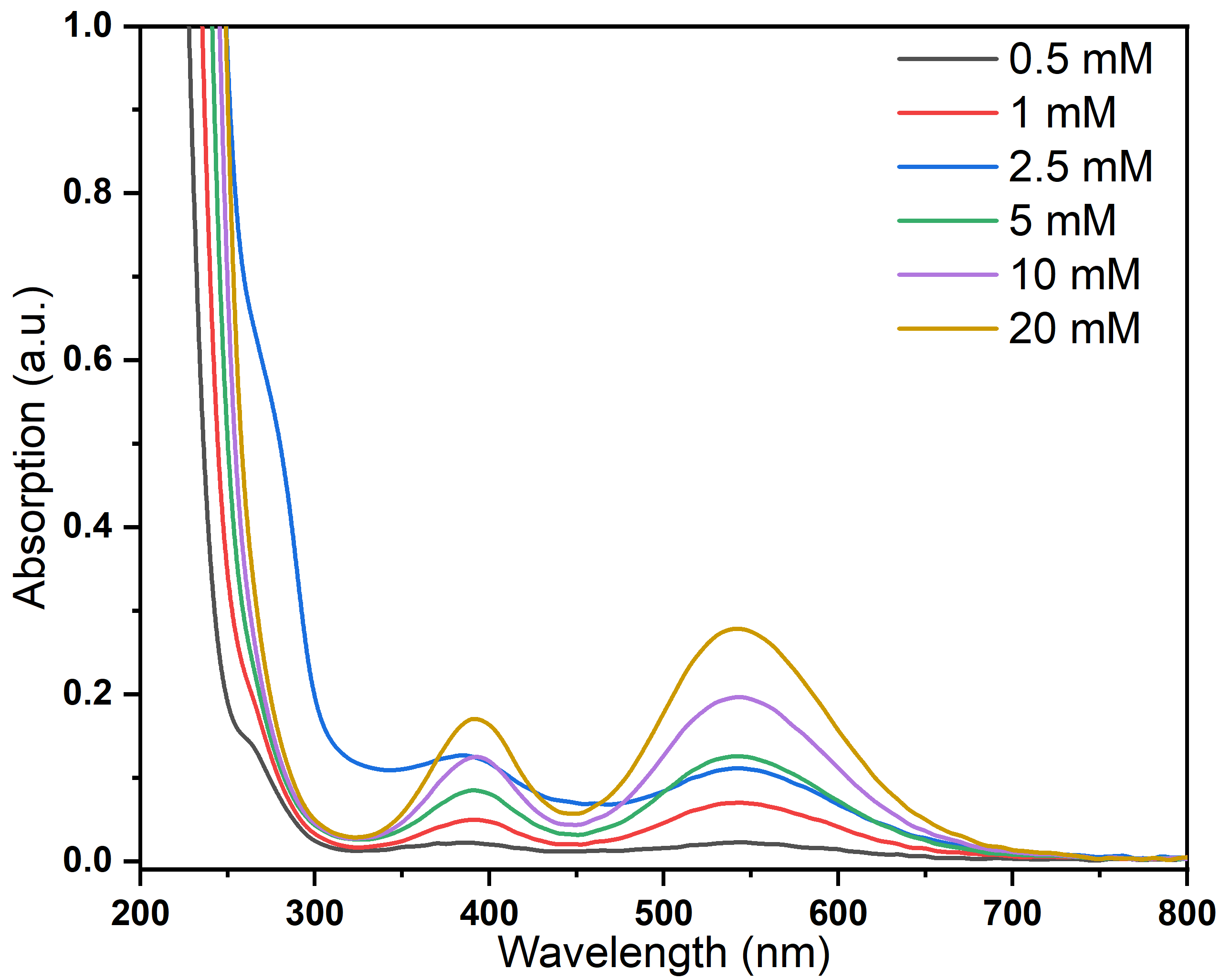


**Figure S18.** The UV-Vis absorption spectra of EDTA and the supernatant from CrGOx with gradient glucose concentrations (0-20 mM).


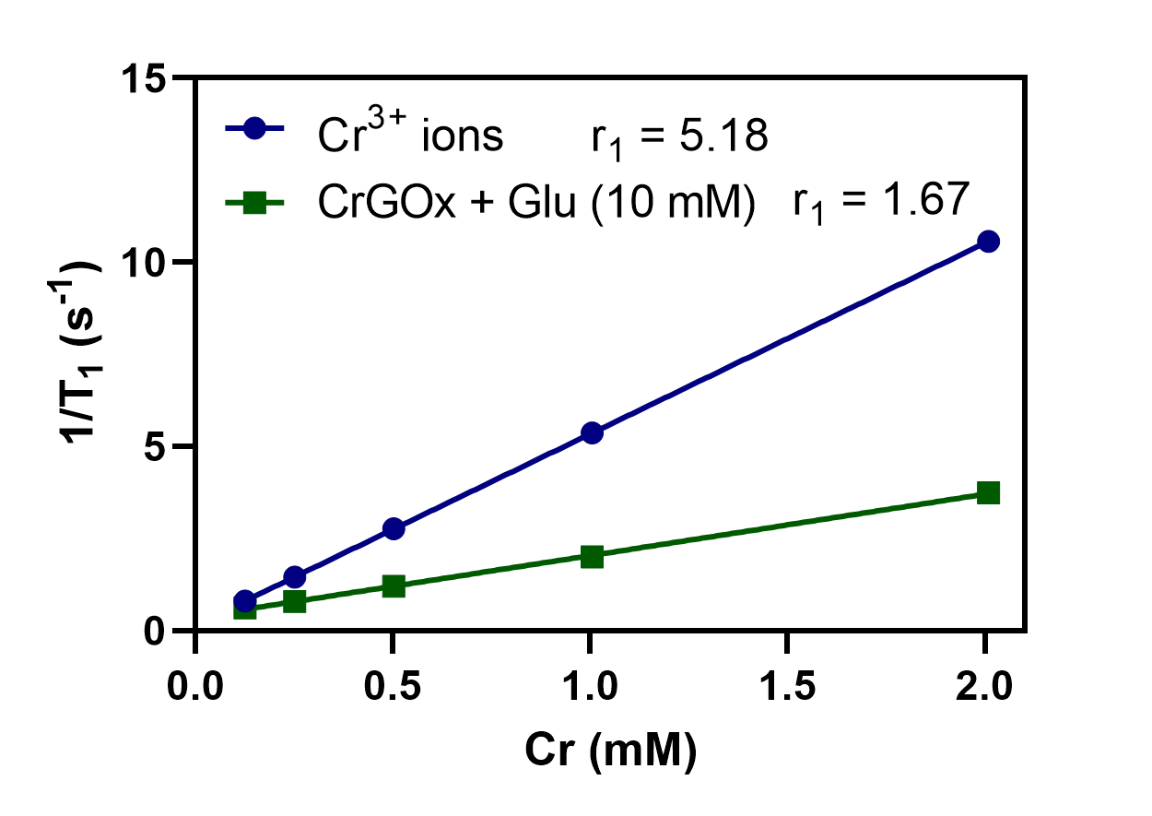


**Figure S19.** The longitudinal relaxation curve fitting of free Cr^3+^ ions and CrGOx

with glucose (10 mM).


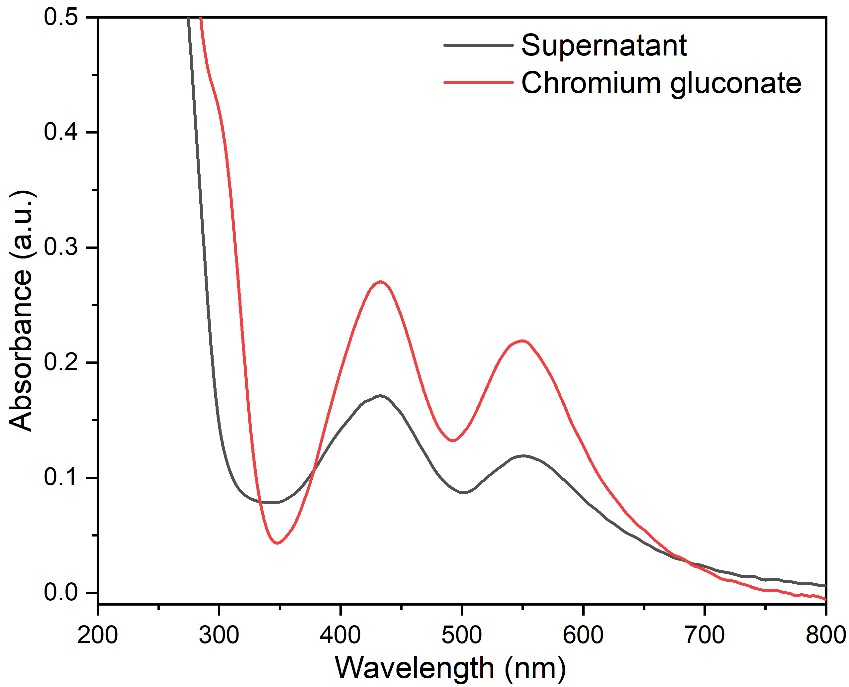


**Figure S20.** The UV-Vis absorption spectra of the supernatant and chromium gluconate standard.


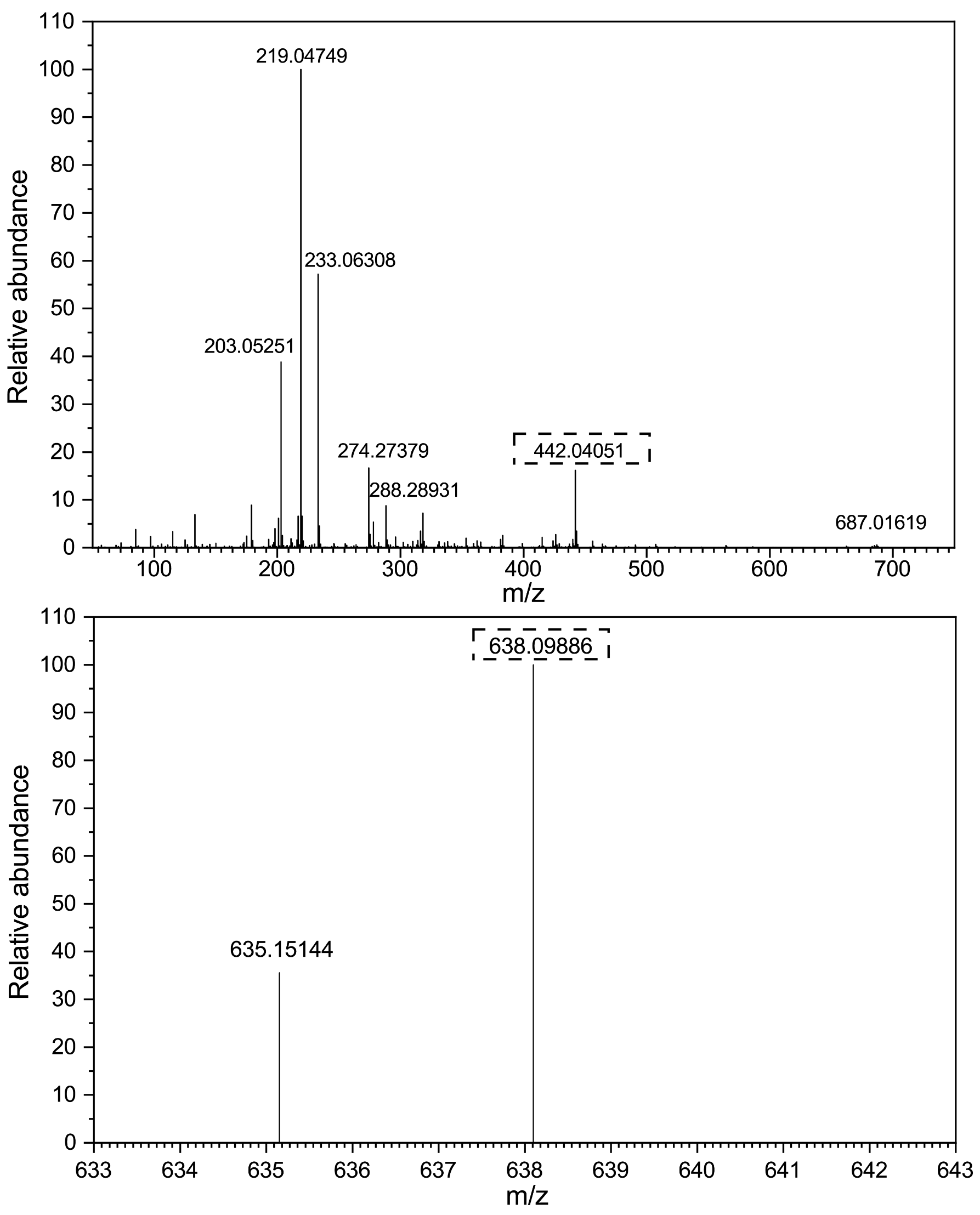


**Figure S21.** Mass spectrum of the supernatant.


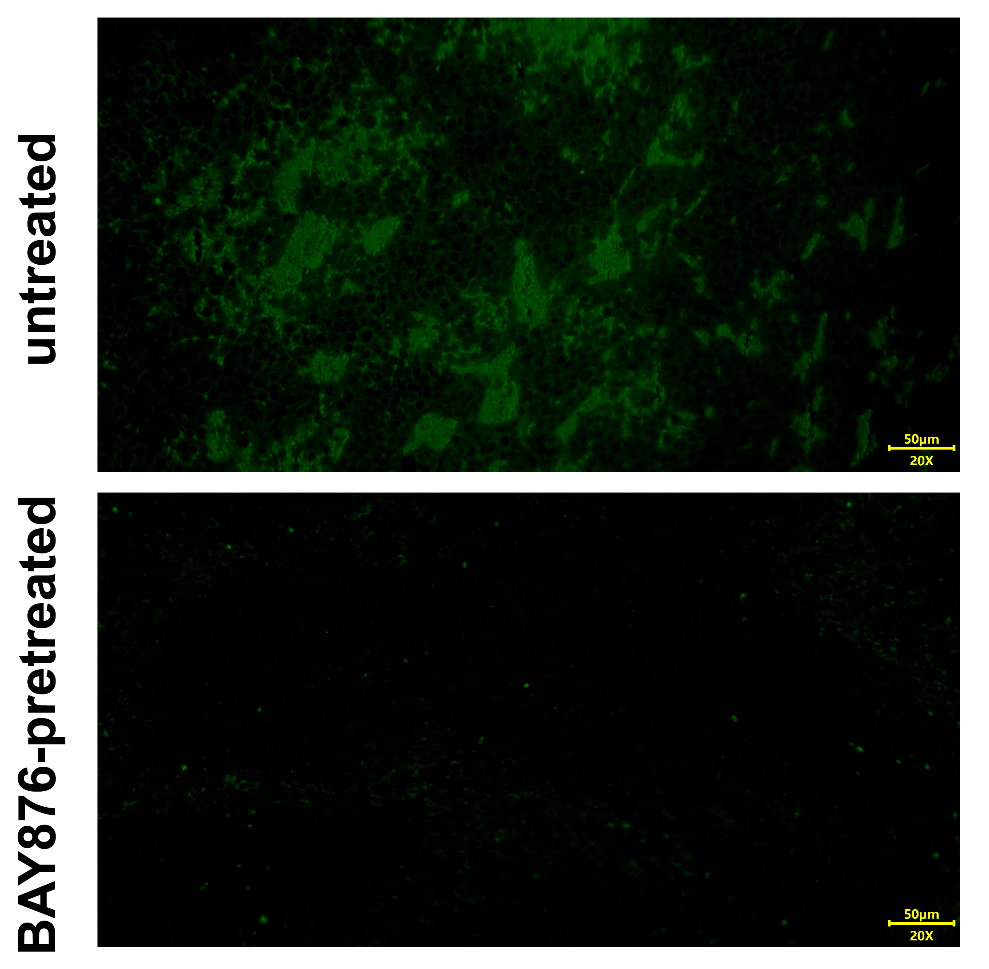


**Figure S22.** Immunofluorescence staining of tumor sections from untreated and BAY876-pretreated mice after CrGOx@Lip injection (scale bar: 50 μm).


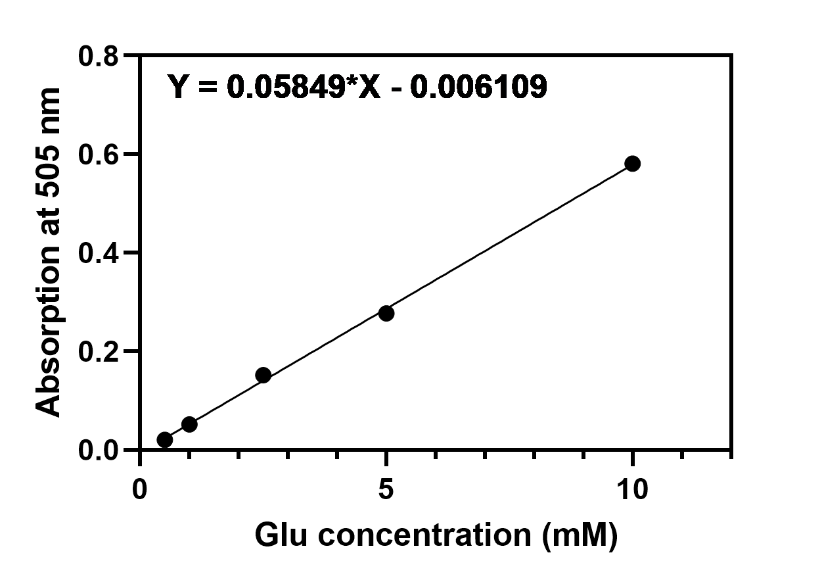


**Figure S23.** The calculated standard curve of glucose tested by glucose assay kit (GOD-POD).


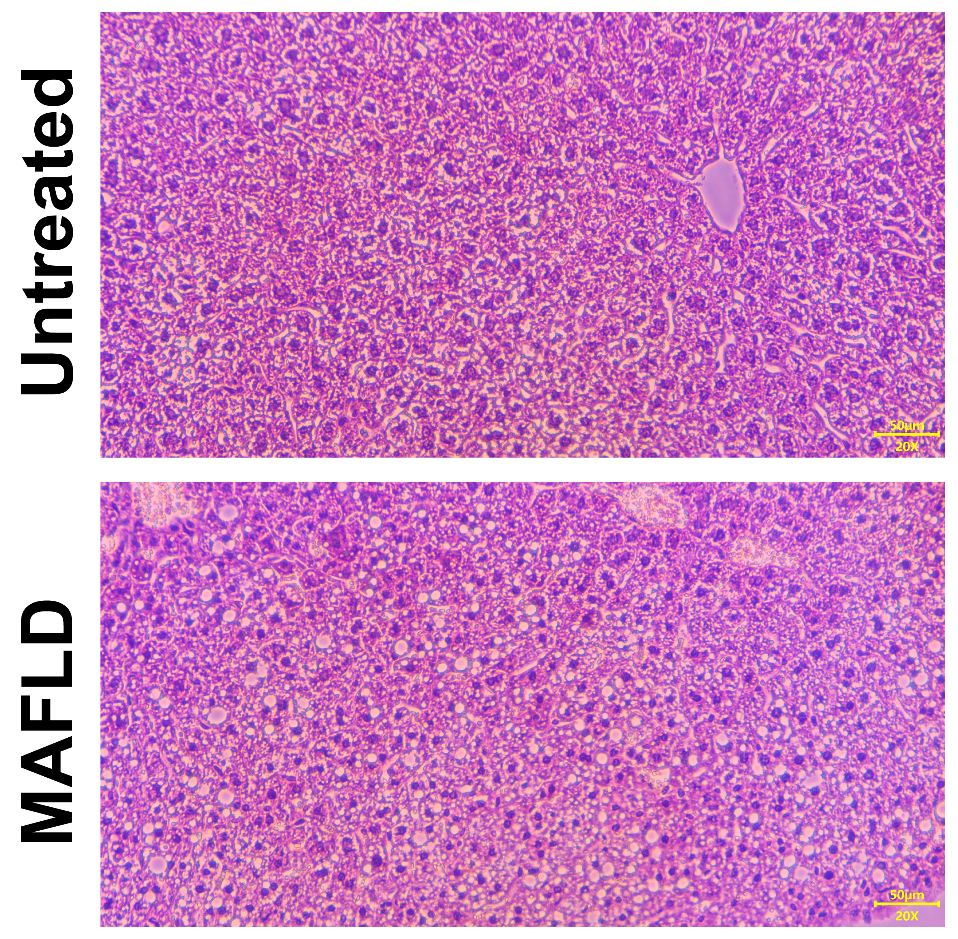


**Figure S24.** H&E staining of livers from untreated mice and high-fat diet induced MAFLD mice (scale bar: 50 μm).


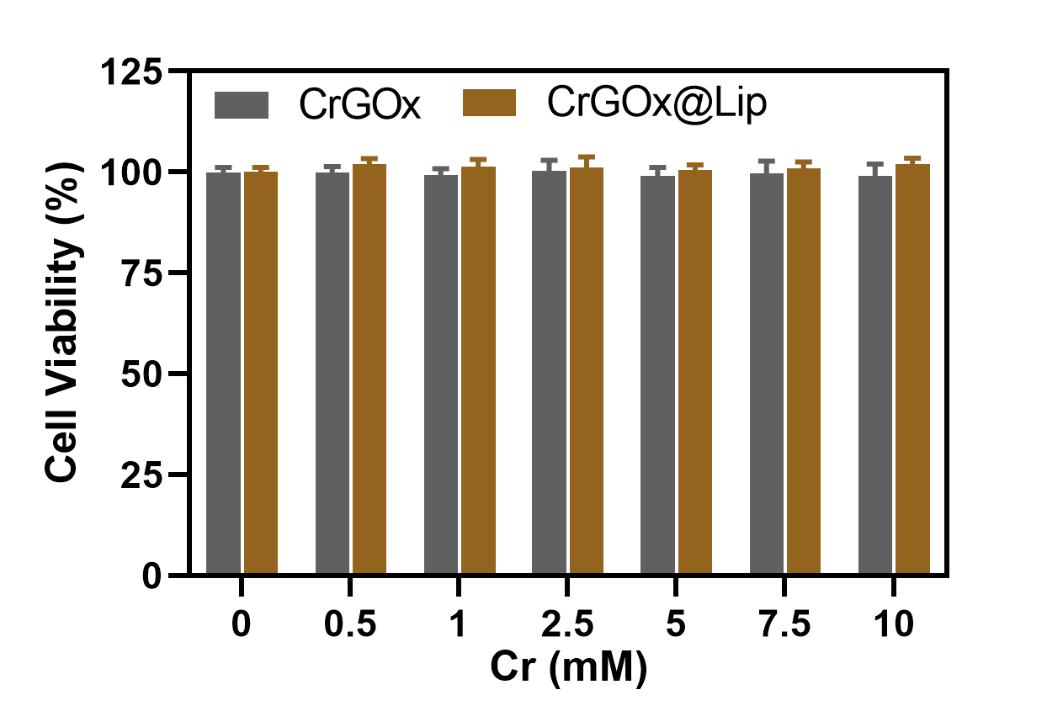


**Figure S25.** The cell viability of L929 cells after incubation with CrGOx or CrGOx@Lip for 24 hours.


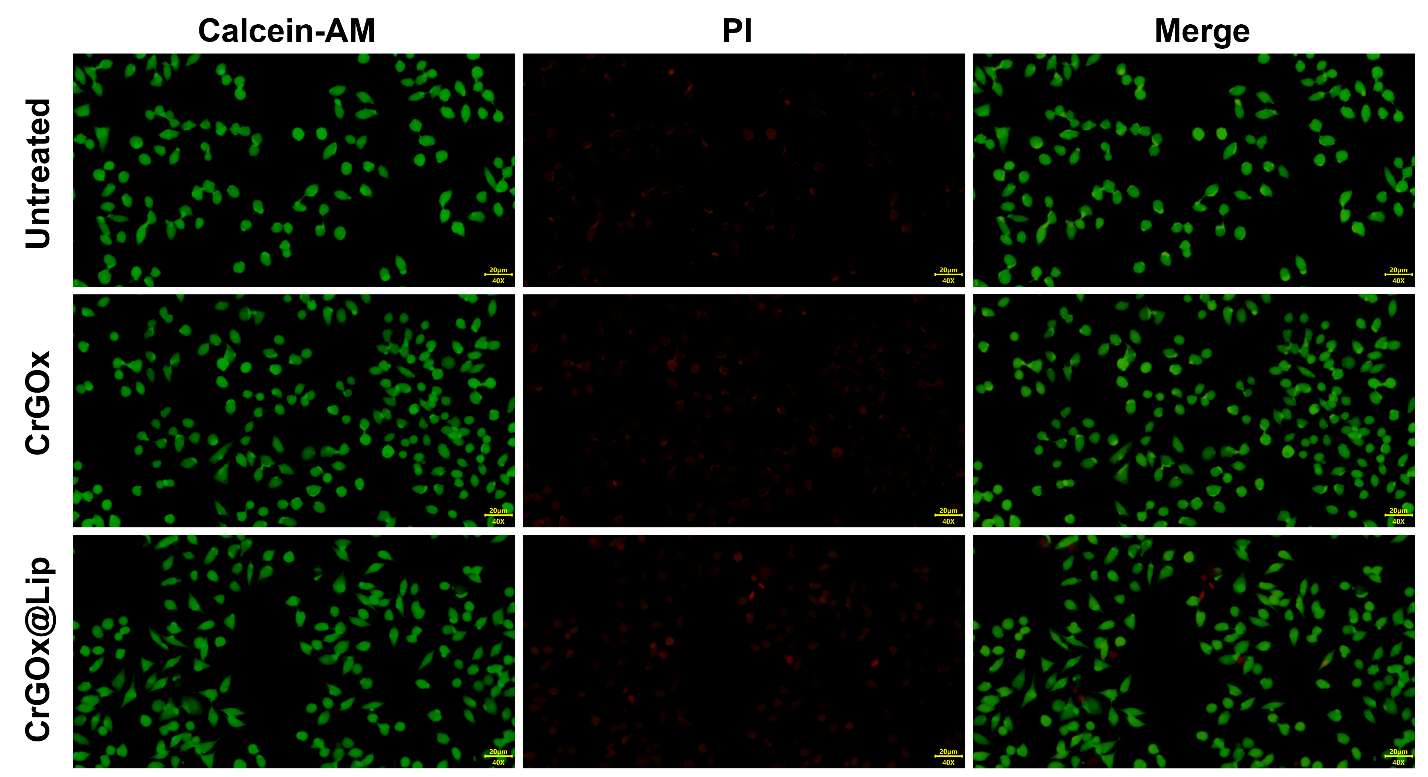


**Figure S26.** Calcein AM/PI staining of L929 cells incubated with CrGOx or CrGOx@Lip for 24 hours (scale bar: 20 μm).


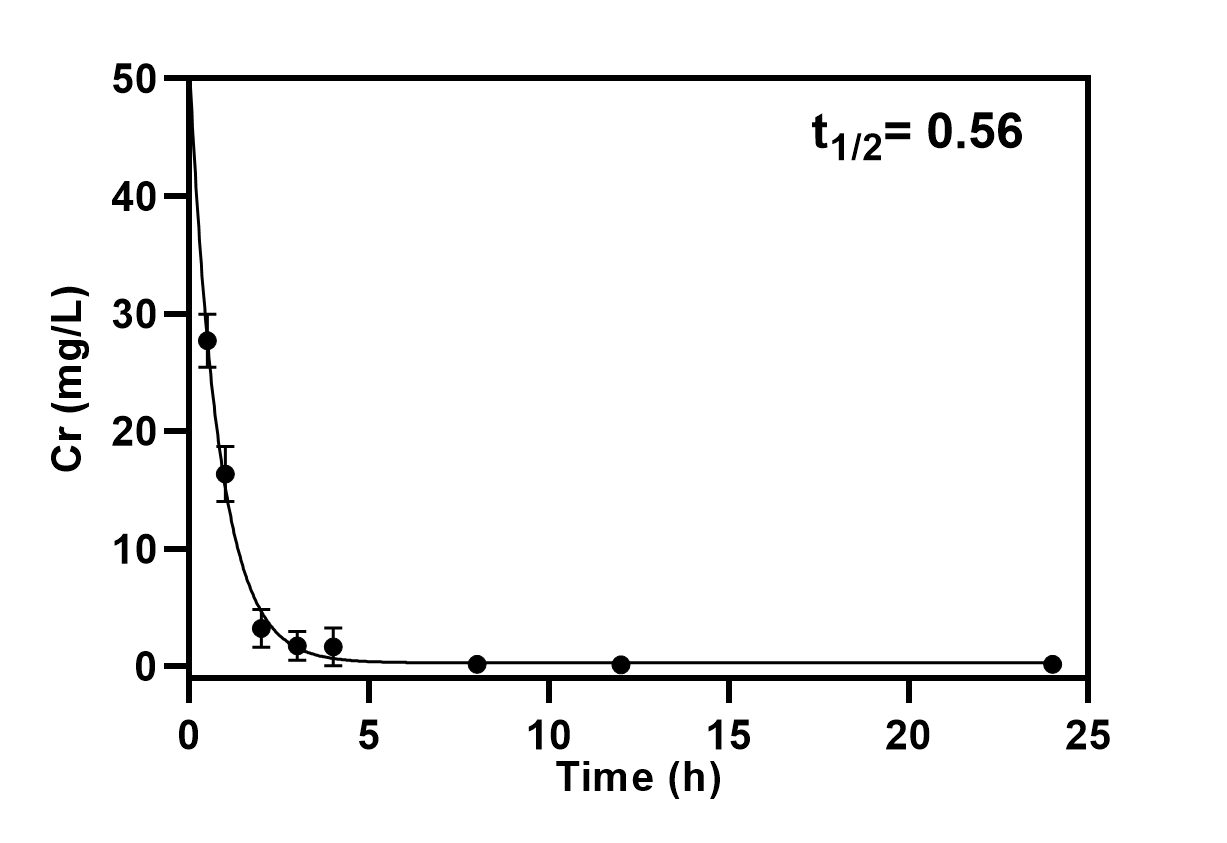


**Figure S27.** Blood circulation half-life analysis for CrGOx@Lip.


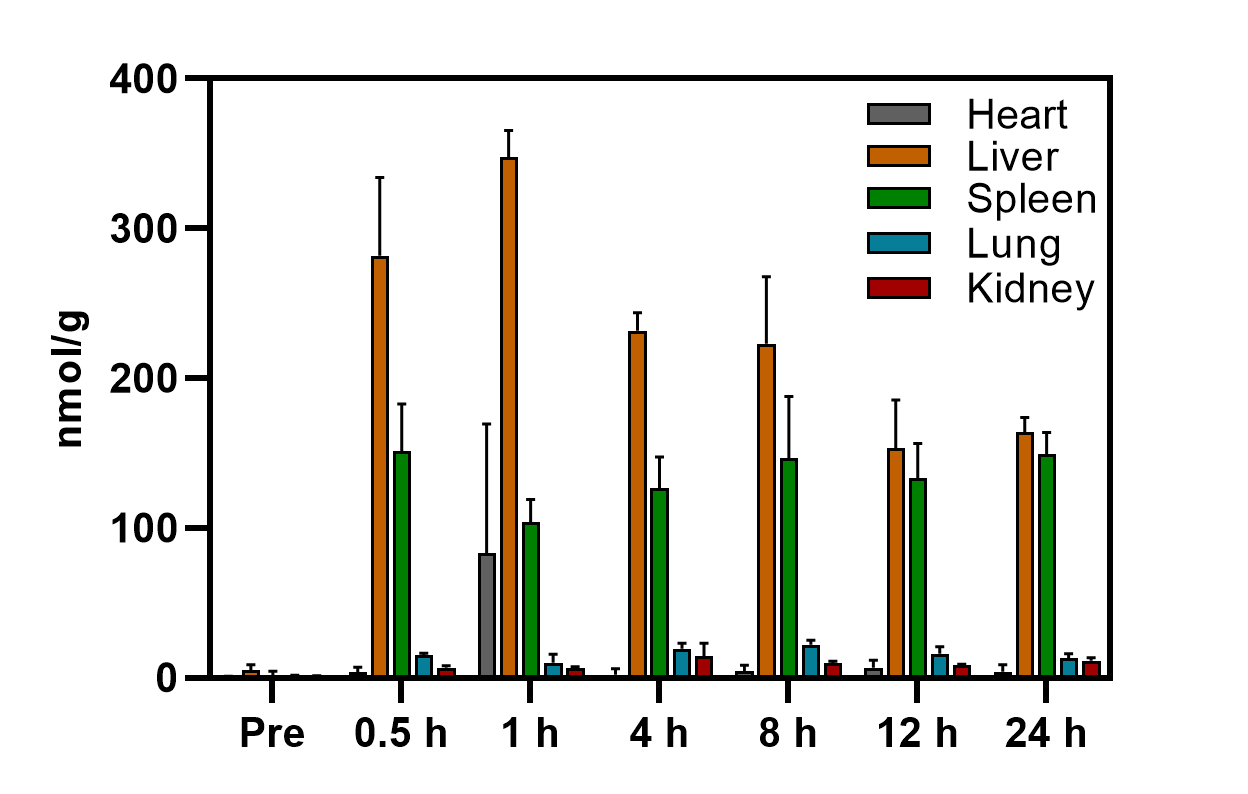


**Figure S28.** The content of chromium in the heart, liver, spleen, lung, and kidney after intravenous administration of CrGOx@Lip.


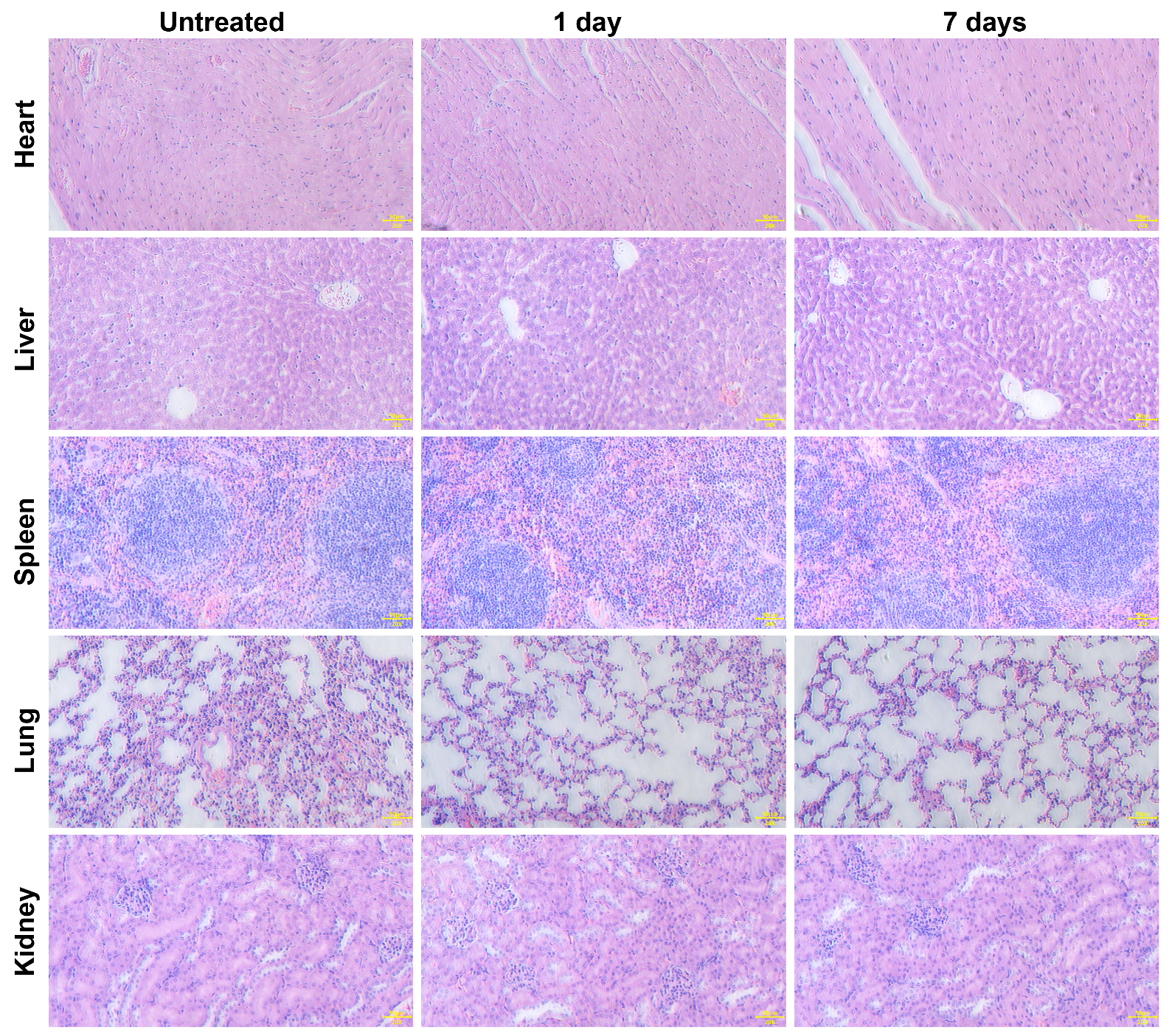


**Figure S29.** H&E staining of hearts, livers, spleens, lungs, and kidneys from normal mice or mice intravenously administrated with CrGOx@Lip (200 µL, 80 mg/kg), and sacrificed after 1 and 7 days (scale bar: 50 μm).

**Table S1.** EXAFS fitting parameters at the Cr K-edge for various samples.

| Sample | Shell | CN^a^ | R(Å)^b^ | σ^2^(Å^2^)^c^ | ΔE_0_(eV)^d^ | R factor |
| --- | --- | --- | --- | --- | --- | --- |
| Cr foil | Cr-Cr | 8.0* | 2.48±0.01 | 0.0066 | 3.5±1.7 | 0.0022 |
|  | Cr-Cr | 6.0* | 2.86±0.01 | 0.0048 |  |  |
| Cr(OH)_3_ | Cr-O | 5.8±0.3 | 1.97±0.01 | 0.0033 | 1.5±1.6 | 0.0119 |
|  | Cr-O-Cr | 5.3±0.5 | 2.95±0.01 | 0.0036 | 6.2±2.8 |  |
|  | Cr-O-Cr | 4.8±0.1 | 3.50±0.01 | 0.0064 | 4.1±3.2 |  |
| CrO_2_ | Cr-O | 6.1±0.3 | 1.92±0.01 | 0.0071 | -5.2±1.8 | 0.0107 |
|  | Cr-O-Cr | 2.3±0.2 | 2.90±0.01 | 0.0046 | -9.9±1.5 |  |
|  | Cr-O-Cr | 5.9±0.5 | 3.45±0.01 | 0.0067 |  |  |
| CrGOx | Cr-O | 5.2±0.2 | 1.98±0.01 | 0.0035 | 0.3±1.3 | 0.0106 |
|  | Cr-O-Cr | 1.3±0.2 | 3.00±0.02 | 0.0043 | -4.1±4.1 |  |
|  | Cr-O-Cr | 0.7±0.3 | 3.64±0.02 | 0.0056 | 17.7±10.6 |  |

Note: (a) CN, coordination number; (b) R, distance between absorber and backscatter atoms; (c) σ2, Debye-Waller factor to account for both thermal and structural disorders; (d) ΔE(0), inner potential correction; R factor indicates the goodness of the fit. S02 was fixed to 0.818 , according to the experimental EXAFS fit of Cr foil by fixing CN as the known crystallographic value. A reasonable range of EXAFS fitting parameters: 0.700 < Ѕ02< 1.000; CN > 0; σ2Å2＞0; |ΔE0|< 15 eV; R factor < 0.02.

**Table S2.** The body weight and tumor volume of mice collected at initial and end point during tumor imaging.

|  | weight before imaging (g) | weight after imaging (g) | tumor volume before imaging (mm^3^) | tumor volume after imaging (mm^3^) |
| --- | --- | --- | --- | --- |
|  | 20.5 | 20.9 | 346.8 | 308.4 |
| CrBSA | 21.2 | 20.5 | 319.4 | 337.0 |
|  | 20.1 | 20.8 | 363.1 | 347.9 |
|  | 19.8 | 20.5 | 346.3 | 365.1 |
| CrGOx | 21.2 | 20.8 | 306.6 | 337.9 |
|  | 20.1 | 21.1 | 334.7 | 362.0 |
|  | 20.7 | 21.2 | 386.6 | 385.8 |
| CrBSA@Lip | 21.5 | 20.9 | 362.4 | 343.2 |
|  | 21.2 | 20.7 | 343.2 | 308.4 |
|  | 22.5 | 21.7 | 324.8 | 343.2 |
| CrGOx@Lip | 20.3 | 21.5 | 340.1 | 353.0 |
|  | 21.5 | 21.1 | 376.5 | 366.5 |
|  | 19.5 | 21.2 | 323.4 | 349.5 |
| BAY876+ CrGOx@Lip | 20.5 | 21.7 | 383.3 | 364.8 |
|  | 19.8 | 21.3 | 327.7 | 309.0 |

**Table S3.** The body weight of MAFLD mice collected at initial and end point during MAFLD glucose imaging.

|  | weight before imaging (g) | weight after imaging (g) |
| --- | --- | --- |
|  | 23.5 | 23.9 |
| Healthy | 25.1 | 24.9 |
|  | 24.5 | 24.8 |
|  | 40.5 | 41.5 |
| MAFLD | 43.2 | 43.5 |
|  | 42.5 | 41.9 |
|  | 41.8 | 42.5 |
| 1 dose | 43.5 | 42.6 |
|  | 40.6 | 40.3 |
|  | 39.8 | 40.5 |
| 3 dose | 42.8 | 43.6 |
|  | 40.2 | 39.5 |
|  | 32.5 | 33.5 |
| 9 dose | 37.8 | 38.6 |
|  | 38.5 | 38.2 |
